# Gene Expression Signature of DMBA-Induced Hamster Buccal Pouch Carcinomas: Modulation by Chlorophyllin and Ellagic Acid

**DOI:** 10.1371/journal.pone.0034628

**Published:** 2012-04-02

**Authors:** Ramamurthi Vidya Priyadarsini, Neeraj Kumar, Imran Khan, Paranthaman Thiyagarajan, Paturu Kondaiah, Siddavaram Nagini

**Affiliations:** 1 Department of Biochemistry and Biotechnology, Faculty of Science, Annamalai University, Tamil Nadu, India; 2 Molecular Reproduction, Development and Genetics, Indian Institute of Science, Bengaluru, India; King Faisal Specialist Hospital & Research Center, Saudi Arabia

## Abstract

Chlorophyllin (CHL), a water-soluble, semi-synthetic derivative of chlorophyll and ellagic acid (EA), a naturally occurring polyphenolic compound in berries, grapes, and nuts have been reported to exert anticancer effects in various human cancer cell lines and in animal tumour models. The present study was undertaken to examine the mechanism underlying chemoprevention and changes in gene expression pattern induced by dietary supplementation of chlorophyllin and ellagic acid in the 7,12-dimethylbenz[a]anthracene (DMBA)-induced hamster buccal pouch (HBP) carcinogenesis model by whole genome profiling using pangenomic microarrays. In hamsters painted with DMBA, the expression of 1,700 genes was found to be altered significantly relative to control. Dietary supplementation of chlorophyllin and ellagic acid modulated the expression profiles of 104 and 37 genes respectively. Microarray analysis also revealed changes in the expression of TGFβ receptors, NF-κB, cyclin D1, and matrix metalloproteinases (MMPs) that may play a crucial role in the transformation of the normal buccal pouch to a malignant phenotype. This gene expression signature was altered on treatment with chlorophyllin and ellagic acid. Our study has also revealed patterns of gene expression signature specific for chlorophyllin and ellagic acid exposure. Thus dietary chlorophyllin and ellagic acid that can reverse gene expression signature associated with carcinogenesis are novel candidates for cancer prevention and therapy.

## Introduction

Chemoprevention by natural products, dietary, and lifestyle changes has evolved as a promising strategy in the management of cancer. Dietary phytochemicals have gained significant recognition in recent years as potential candidates for cancer chemoprevention owing to their ability to arrest or reverse the cellular and molecular processes associated with carcinogenesis [1]. Chlorophyllin (CHL), a water-soluble, semi-synthetic derivative of chlorophyll, and ellagic acid (EA), a naturally occurring polyphenolic compound in berries, grapes, and nuts have been reported to exert potent antimutagenic and anticarcinogenic effects [2]–[4].

The anticarcinogenic effects of chlorophyllin have been attributed to its ability to scavenge reactive oxygen species and form complexes with planar carcinogens [2]. The anticancer activity of chlorophyllin first demonstrated in the rainbow trout was subsequently documented in several animal tumor models [5]. Chlorophyllin has also been reported to exhibit antiproliferative effects in colon, breast, and leukemic cancer cell lines [6]–[8]. Human intervention trials with chlorophyllin showed a decrease in aflatoxin-DNA adducts in individuals at high risk for liver cancer [9].

Dietary ellagic acid either independently or synergistically is responsible for a plethora of health beneficial effects. Ellagic acid displays antimutagenic and anticarcinogenic effects against a variety of carcinogens including nitrosamines, azoxymethane, mycotoxins, and polycyclic aromatic hydrocarbons [10]–[13]. Ellagic acid also exerts anticancer effects in various human cancer cell lines and in animal tumour models and has been found to function as both a blocking and suppressing agent in carcinogenesis [14]–[18].

Despite the profuse epidemiological data, and studies on cell and animal models, the mechanism underlying chemoprevention and changes in gene expression pattern induced by chlorophyllin/ellagic acid has remained largely unexplored. The present study was undertaken to identify the genes that are differentially modulated by treatment with chlorophyllin/ellagic acid during 7,12-dimethylbenz[a]anthracene (DMBA)-induced hamster buccal pouch (HBP) carcinogenesis, an ideal animal model for analyzing the effects of putative chemopreventive agents [19]. To this end, we examined whole-genome expression using pangenomic microarrays to unveil the molecular mechanisms and signaling pathways regulated by chlorophyllin and ellagic acid.

## Results

### Body weight, tumour incidence, and histopathological changes

**Figure 1:**
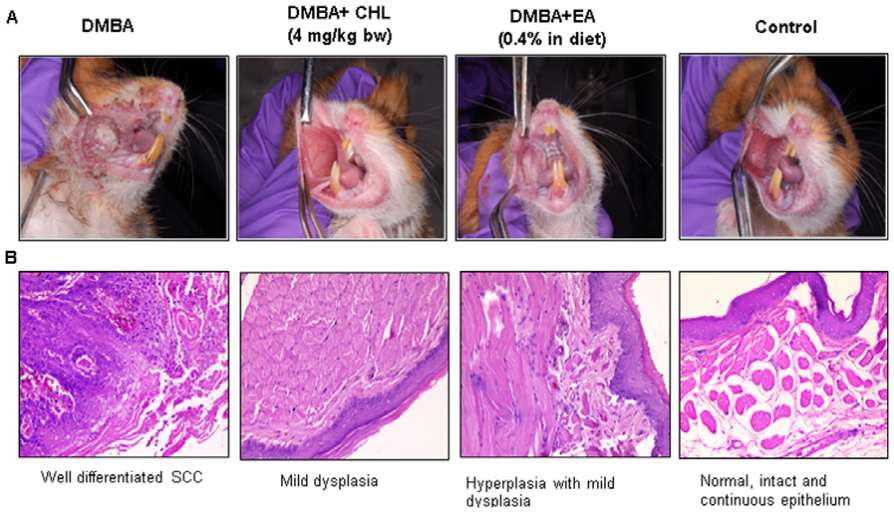
Gross appearance (A) and photomicrographs of histopathological changes (B) in the buccal pouch mucosa of control and experimental animals (×20).

The mean final body weights were significantly decreased in group 1 (DMBA) compared to control (group 4). No significant differences in the body weights were observed in group 2 and 3 animals. In group 1 animals, the incidence of SCC was 100% with a tumour multiplicity of 2.6 per hamster. These tumours were large and exophytic with a mean tumour burden of 77.32 mm^3^. No tumours were observed in group 2 (DMBA+chlorophyllin) and group 3 (DMBA+ellagic acid) animals. Histopathological examination of these pouches revealed mild to moderate hyperplasia. In group 4 animals, the epithelium was normal, intact, and continuous. The gross appearance and representative photomicrographs of histopathological changes in the buccal pouch mucosa of control and experimental animals are shown in Figure 1.

### Differentially expressed genes

**Figure 2:**
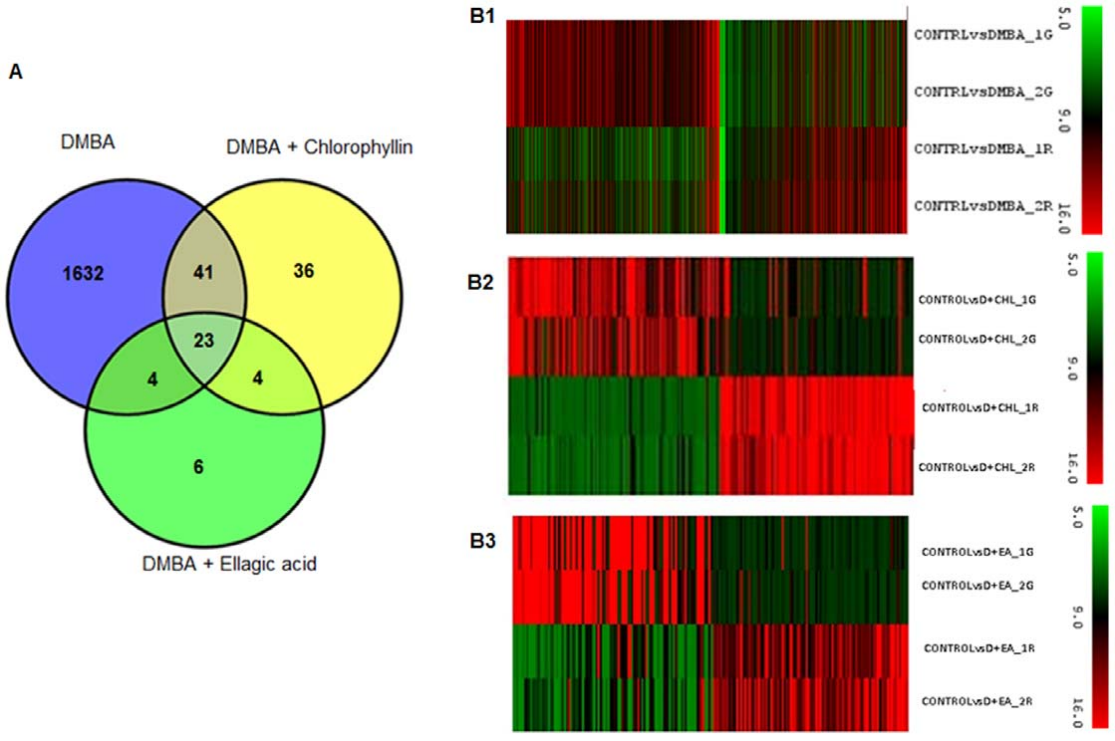
Gene expression profiles in various experimental groups. (A) Venn diagram analysis of the differentially expressed genes with P value = 0.05 and two fold change cut off in various experimental groups. (B) Heat maps depicting the gene expression profiles of hamsters treated with DMBA (B1), DMBA+chlorophyllin (B2) and DMBA+ellagic acid (B3). The heat map represents unsupervised clustering of differentially-expressed genes (P value = 0.05, fold change cut off of 2) using Imfit and eBayes (Empirical Bayes) method. Heat map values are log_2_-transformed, normalized fluorescence ratios of control versus treatment groups. Red represents an expression level above the mean expression of a gene across all samples; black represents mean expression; green indicates expression lower than the mean.

Analysis of microarray data revealed differential expression of 13,277 genes in DMBA painted hamsters relative to control. In hamsters supplemented with chlorophyllin and ellagic acid in the diet, 3,899 and 1,420 genes were differentially expressed relative to control. Using a P value = 0.05 and fold change cut off of 2, differentially expressed genes were identified between DMBA, DMBA+chlorophyllin, DMBA+ellagic acid groups by Imfit and eBayes (Empirical Bayes method). We found that 1,700 genes were differentially expressed in DMBA (Table S1); 104 genes in DMBA+chlorophyllin (Table S2); and 37 genes in DMBA+ellagic acid treated groups relative to control (Table S3). Venn diagram analysis of the differentially regulated genes indicated that 64 genes were commonly regulated in DMBA and DMBA+chlorophyllin, and 27 genes in DMBA and DMBA+ellagic acid groups. Furthermore, 27 genes were identified to be universally regulated by both chlorophyllin and ellagic acid (Figure 2). The microarray data has been deposited in NCBI's Gene Expression Omnibus (GEO) with GEO series accession number GSE29679. Heat map diagrams of differentially expressed genes in DMBA, DMBA+chlorophyllin, and DMBA+ellagic acid groups are shown in Figure 2.

### Hierarchical clusters and molecular pathways modulated

Gene enrichment analysis using GSEA Pre Ranked Tool revealed that 67 pathways were significantly dysregulated in DMBA painted hamsters. The major functional categories of the up-regulated genes in DMBA painted animals were associated with cell cycle, TGFβ signalling, and other signal transduction cascades, whereas the most significantly downregulated gene functions were associated with cell adhesion and apoptosis (Table 1). Dietary administration of chlorophyllin and ellagic acid were found to universally modulate expression of genes associated with cell adhesion, cell-cell communication, and TGFβ signaling (Table 2).

**Table 1:** Molecular pathways related to the genes differentially expressed in DMBA painted animals.

Functional classification	Genes Symbols
***DMBA- upregulated***	
HSA04110_Cell cycle	CCNB1, CCND1, CCND3, TGFB1, PCNA, CDK7
HSA04512_ECM receptor interaction	COL5A3, COL5A1, THBS4, CD44, TNR
HSA05219_Bladder cancer	MMP9, MMP2, EGFR
HSA04630_JAK STAT signaling pathway	JAK2, PTPN6, STAT1, CCND3
HSA04670_Leukocyte trans endothelial migration	MMP9, MMP2, ACTN1, CYBA, CXCL12
HSA04510_Focal adhesion	COL5A3, EGFR, PDGFRB, MAPK8, CCND1, JUN, TNR, SRC
HSA05222_Small cell lung cancer	CCNE1, NOS3, CCND1, NFKB1
HSA04350_TGF beta signaling pathway	TGFB1, SMAD7, THBS4
***DMBA- downregulated***	
HSA03320_PPAR signaling pathway	FABP3, HMGCS2, ACSL6, ME1
HSA04530_Tight junction	MYH2, MYH6, OCLN, PPP2R2A, PRKCH, F11R, PARD3
HSA04520_Adherens junction	LMO7, PARD3, ACTN4, ERBB2
HSA04514_Cell adhesion molecules	NRXN1, OCLN, CDH15, CLDN1, L1CAM
HSA04210_Apoptosis	CAPN1, PIK3R1, PPP3CA, PRKAR1A, CHP

Gene enrichment analysis for the up- and down-regulated genes was done using GSEA Pre Ranked Tool. The selection criteria were set at Benjamin-Hochberg P = 0.05. Only statistically significant pathways showing a permuted P-value = 0.05 and a positive (enrichment) z-score >2 were selected.

**Table 2:** Molecular pathways related to the genes commonly modulated by chlorophyllin and ellagic acid supplementation.

Functional classification	Genes Symbols
***Chlorophyllin/Ellagic acid- upregulated***	
HSA04514_Cell adhesion molecules	NRCAM, CLDN1, NRXN1
HSA03320_PPAR signaling pathway	UBC, HMGCS2
Hsa04080_Neuroactive ligand receptor interaction	OPRM1, P2RX2, GLP1R, EDG5, DRD4
***Chlorophyllin/Ellagic acid- downregulated***	
HSA04350_TGF beta signaling pathway	TGFB1
Hsa03050_Proteasome	PSMA7

Genes commonly modulated by chlorophyllin and ellagic acid in the enriched pathways (z-score >2) are listed. Gene enrichment analysis was done using GSEA Pre Ranked Tool. P value correction was done using Benjamini and Hochberg method.

### Validation of microarray results by qPCR and Western blot analysis

**Figure 3:**
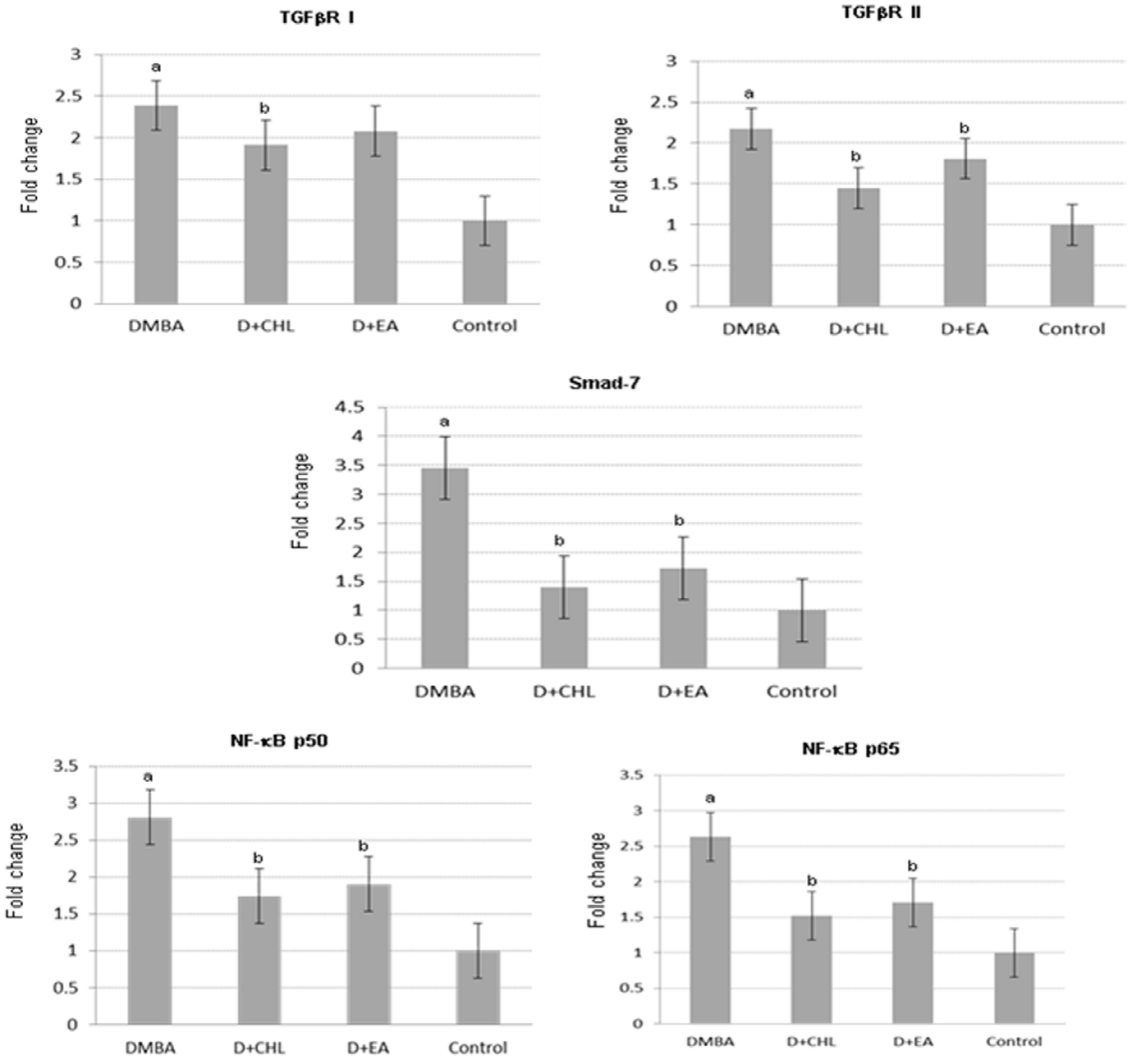
Transcript expression levels of TGFβRI, TGFβRII, Smad7, NF-κB p50, and NF-κB p65 in various experimental groups as determined by RT-qPCR. cDNA individually generated from the buccal pouch RNA of biological replicates was subjected to RT-qPCR analysis. Relative mRNA expression for each gene was determined and normalized to the average transcript expression of GAPDH internal control. The fold change in transcript expression for each gene was determined using the 2^−ΔΔCt^ method. Data are the mean ± SD of two separate experiments. Statistical significance was determined by Mann-Whitney test (P<0.05). a Significantly different from control. b Significantly different from DMBA.

**Figure 4:**
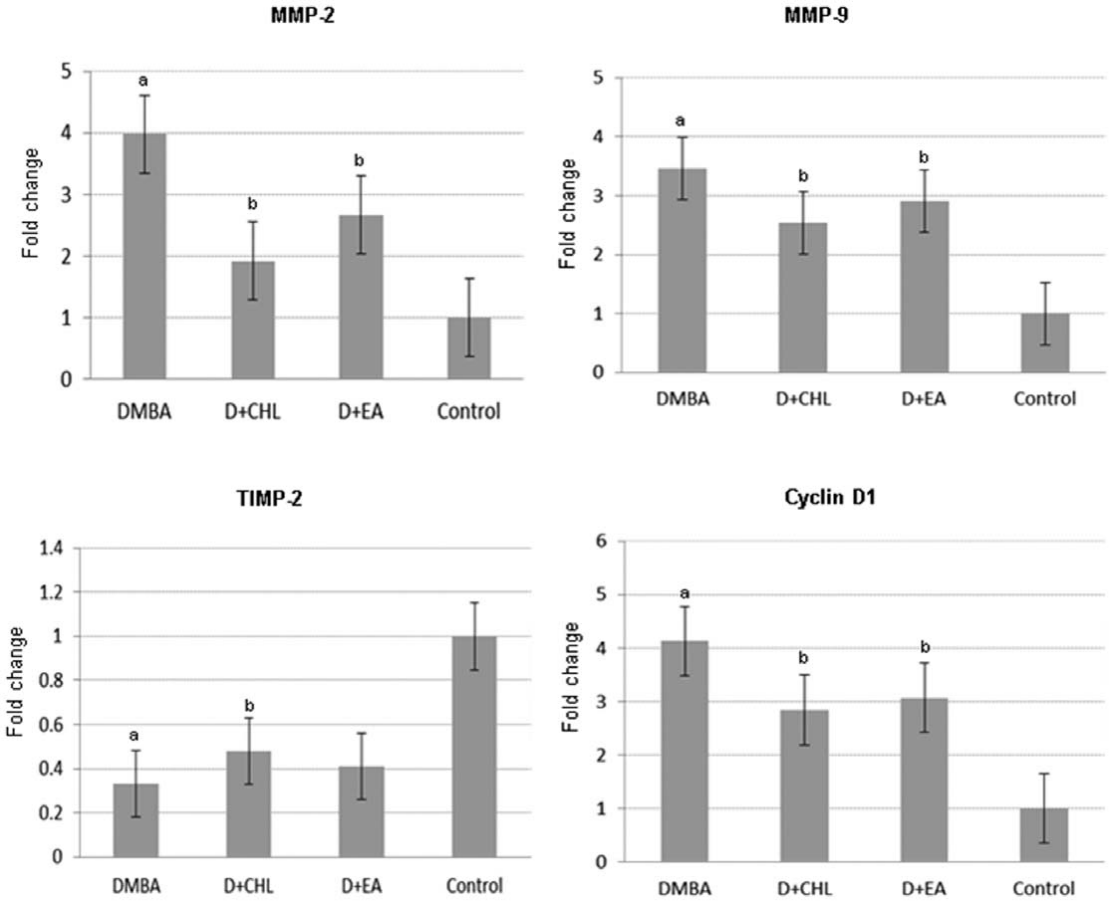
Relative mRNA expression levels of MMP-2, MMP-9, TIMP-2 and Cyclin D1 in various experimental groups as determined by RT-qPCR. cDNA individually generated from the buccal pouch RNA of biological replicates was subjected to RT-qPCR analysis. Relative mRNA expression for each gene was determined and normalized to the average transcript expression of GAPDH internal control. The fold change in transcript expression for each gene was determined using the 2^−ΔΔCt^ method. Data are the mean ± SD of two separate experiments. Statistical significance was determined by Mann-Whitney test (P<0.05). a Significantly different from control. b Significantly different from DMBA.

**Figure 5:**
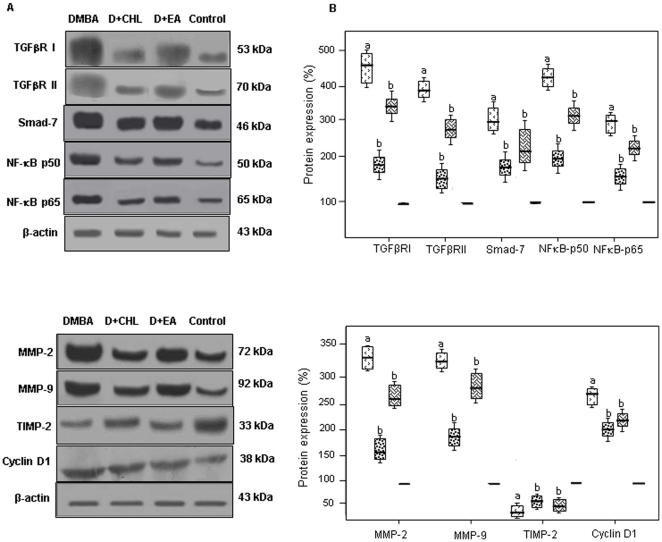
Western blot analysis of the differentially expressed genes. (A) Representative immunoblots showing the protein levels of the differentially expressed genes in the buccal pouch total cell lysate. Protein samples (50 µg/lane) resolved on SDS-PAGE were probed with corresponding antibodies. Quantification was done by normalising the band density to that of β-actin. (B) Densitometric analysis. Each box plot represents the protein expression from control lysates for two determinations. a Significantly different from control by Mann-Whitney test (P<0.05). b Significantly different from DMBA.

We performed quantitative reverse transcriptase PCR (qRT-PCR) and western blot analysis to validate the results of microarray analysis (Figures 3, 4, 5). A significant increase in the expression of TGFRI, TGFRII, Smad7, NF-κB p50, NF-κB p65, MMP-2, MMP-9, and cyclin D1, with decrease in the expression of TIMP-2 was found in DMBA painted pouches compared to control. Dietary supplementation of chlorophyllin and ellagic acid to DMBA painted hamsters significantly downregulated both the mRNA and protein expression of TGFRII, Smad7, NF-κB, MMP-2, MMP-9, and cyclin D1 compared to group 1 animals confirming the results of microarray analysis. Although immunoblot analysis revealed significant modulatory effects of both the phytochemicals on protein expression of TGFRI and TIMP-2, the qRT-PCR data indicated significant changes only for the chlorophyllin supplemented group.

## Discussion

A substantial number of studies have demonstrated the chemopreventive efficacy of chlorophyllin and ellagic acid in various animal tumour models [16]–[18], [20], [21]. We report for the first time the inhibition of DMBA-induced HBP carcinogenesis by dietary supplementation of chlorophyllin and ellagic acid based on reduced incidence of preneoplastic and neoplastic lesions, and changes in the expression pattern of genes associated with carcinogenic signaling.

Comparative gene expression profiling revealed differential expression of a substantial number of gene transcripts in HBP tumours with a fold change greater than 2. Dietary administration of chlorophyllin and ellagic acid significantly reversed the expression of a subset of differentially expressed genes in DMBA painted animals towards normal, suggesting that these genes may be responsible for the chemopreventive potential of these phytochemicals. In particular, downregulation of TGFβ receptors, Smad7, MMPs, cyclin D1, and NF-κB by chlorophyllin and ellagic acid may play a key role in impeding the development of HBP tumours.

Of late, TGF-β has emerged as a promising therapeutic target against cancer and several small and large-molecule compounds that block TGF-β signalling have entered clinical trials [22], [23]. TGF-β, a multifunctional cytokine regulates a diverse array of cellular processes including morphogenesis, motility, differentiation, cell proliferation, apoptosis, and invasion in various cell types. TGF-β signalling is mediated through two serine/threonine kinase transmembrane receptors, TβRI and RII that activate Smads. Activated Smads relocate to the nucleus and together with other transcription factors regulate the expression of target genes [24]. TGF-β plays a dual role in oncogenesis, functioning as a tumor suppressor in early stages of tumour development and as an oncogene in later stages [25]. Downregulation of TβRI and RII as well as Smad-7 by chlorophyllin and ellagic acid supports the view that tumors in which TGF-β functions as an oncogenic factor are good candidates for anti-TGF-β therapy.

Modulation of TGFβ signaling by chlorophyllin and ellagic acid was associated with downregulation of nuclear factor-kappa B (NF-κB). Strategies that target NF-κB, a hub in oncogenic signaling are considered central in designing effective anticancer agents [26], [27]. NF-κB, a redox-sensitive transcription factor functions as a major mediator of inflammation, cell cycle progression, cell survival, and metastasis during carcinogenesis. In unstimulated cells, NF-κB exists in the cytosol as an inactive heterodimer of p50 and p65 subunits complexed to inhibitor of NF-κB (IκB). Upon stimulation, IκB undergoes rapid phosphorylation by IκB kinase β (IKKβ) and degradation via the ubiquitin proteasome pathway. Dissociation of IκB from the p50/p65 heterodimer exposes the nuclear localization signals on NF-κB that subsequently translocates to the nucleus and regulates the transcription of over 500 genes implicated in oncogenesis [28], [29]. In particular, transactivation of cyclin D1 and epithelial-mesenchymal transition (EMT) programming genes such as matrix metalloproteinases (MMPs) by NF-κB is considered critical for malignant transformation [30]–[32].

Cyclin D1, an oncogene that promotes cell cycle progression through phosphorylation of the retinoblastoma protein and prevents apoptosis by sequestering Bax in the cytoplasm has been recognized as a potential therapeutic target in cancer [33], [34]. Downregulation of cyclin D1 by dietary supplementation of chlorophyllin and ellagic acid may be attributed to abrogation of signaling through receptor tyrosine kinases, and the MEK–ERK, WNT, and NF-κB pathways as well as TGF-β mediated antiproliferative control [34].

The results of the present study suggest that deregulation of TGFβ/NF-κB signaling triggers upregulation of MMP-2 and -9 in HBP carcinomas. Numerous studies have demonstrated a direct link between TGFβ/NF-κB signaling and MMP overexpression in various cancers [35]–[38]. MMPs, a family of zinc-dependent endopeptidases that catalyze the disintegration of the ECM and basement membrane play a pivotal role in tumor invasion and angiogenesis [39]. Despite the limited success of MMP inhibitors in clinical trials, several pharmaceutical companies have invested considerable efforts to develop safe and effective agents to target MMPs [40]. Chlorophyllin and ellagic acid that decrease the expression of MMP-2 and -9 are attractive candidates for inhibiting MMPs and consequent tumor progression.

In summary, our results reveal multiple gene changes in DMBA-induced HBP carcinomas. In particular, changes in the expression of TGFβ receptors, NF-κB, cyclin D1, and MMPs play a crucial role in the transformation of the normal buccal pouch to a malignant phenotype. Consistent with our findings, Yang et al. [41] reported that 5,255 genes were differentially expressed during the development and progression of HBP carcinomas most of which were associated with cell structure, cell adhesion, cell differentiation, signal transduction, and transcription regulation. The ability of chlorophyllin and ellagic acid to modulate the expression of these genes supports the notion that these phytochemicals acid display chemopreventive efficacy by reversing gene expression signature associated with tumorigenesis. Our study has also revealed patterns of gene expressions that offer clues for both the chemopreventive mechanism as well as molecular gene expression signature specific for chlorophyllin and ellagic acid exposure.

Over the last several years there has been an increasing focus on developing anticancer agents that target multiple molecules or pathways that are aberrant in cancer. Recent studies from this laboratory and by others have demonstrated that ellagic acid and chlorophyllin exert their anticancer effects by blocking DNA damage, WNT and NF-κB signaling, and inducing apoptosis [42]–[44]. Taken together our results indicate that dietary chlorophyllin and ellagic acid that can simultaneously target several dysregulated pathways and multiple molecules with pleiotropic effects and reverse gene expression associated with carcinogenesis are novel candidates for cancer prevention and therapy.

## Materials and Methods

### Chemicals

Chlorophyllin, ellagic acid, acrylamide, bovine serum albumin (BSA), bromophenol blue, diethylpyrocarbonate (DEPC), DMBA, 2-mercaptoethanol, sodium dodecylsulphate (SDS), N,N,N′,N′-tetramethylene diamine (TEMED), and trizol were purchased from Sigma Chemical Company, St. Louis, MO, USA. All other reagents used were of analytical grade. Power SYBR® Green PCR master mix was purchased from Applied Biosystems, California, USA. Oligonucleotide primers were purchased from Sigma Genosys, San Ramon, USA. Primary and secondary antibodies were purchased from Santa Cruz Biotechnology, USA.

### Animals and diet

The experiment was carried out with male Syrian hamsters aged 8–10 weeks weighing 100–110 g obtained from the Central Animal House, Annamalai University, India. The animals housed four to a polypropylene cage were provided with standard pellet diet (Kamadhenu Agencies, Bengaluru, India) and water *ad libitum* and maintained under controlled conditions of temperature and humidity with an alternating light/dark cycle in accordance with the guidelines of the Indian Council of Medical Research, and approved by the ethical committee, Annamalai University (approval ID-670).

### Experimental design

The animals were randomized into experimental and control groups and divided into 4 groups of eight animals each. In group 1, the right buccal pouches of hamsters were painted with a 0.5 per cent solution of DMBA in liquid paraffin using a No.4 sable brush, three times per week for 14 weeks as described earlier [45]. Group 2 and 3 animals painted with DMBA as in group 1, received 4 mg/kg bw of chlorophyllin and 0.4% of ellagic acid respectively in the diet for 14 weeks. Group 4 animals received basal diet and served as control. The doses of chlorophyllin and ellagic acid used in this study were chosen based on a dose response study undertaken by us that demonstrated maximum chemopreventive efficacy at these doses as well as on previous reports [9], [42], [46], [47]. The experiment was terminated at the end of 14 weeks and all animals were sacrificed by cervical dislocation after an overnight fast.

### RNA extraction, microarray hybridization and data analysis

Total RNA was isolated from the buccal pouch of two individual hamsters in each experimental group using Trizol as described previously [48]. Isolated RNA was then purified using RNAeasy columns (Qiagen GmbH, Hilden, Germany) and treated with ribonuclease free DNaseI to remove any contaminating DNA. RNA was quantitated by NanoDrop ND-1000 (Thermo Scientific, Wilmington DE) and the quantity and integrity was established by resolving on 0.8% formaldehyde agarose gels.

Microarray experiments were performed in replicates using Agilent Whole Rat Genome Oligo Microarray Kit, 4×44 k. As genome sequence information of hamsters are relatively limited, rodent microarrays such as mouse or rat DNA microarrays are used for the transcriptome profiling of Syrian hamster tissues and Chinese hamster ovary cells [49]. For the microarray labeling reaction, 200 ng of isolated RNA from each sample was used. Labeling was done using the Low Input Quick Amp Labeling Kit (Agilent Technologies, Santa Clara, CA) as per the manufacturer's protocol. Briefly, using affinity script-RT oligo dT promoter primer, cDNA was generated and from the cDNA, labeled cRNA was generated via an *in vitro* transcription reaction using T7 RNA polymerase and Cy5 (DMBA, DMBA+chlorophyllin, DMBA+ellagic acid) or Cy3 (control) CTP. Labeled cRNA (825 ng) from each sample was used for hybridization in the following combinations- DMBA (Cy5) and control (Cy3), DMBA+chlorophyllin (Cy5) and control (Cy3) and DMBA+ellagic acid (Cy5) and control (Cy3). Hybridization was performed for 17 h, rotating at a speed of 10 rpm at 65°C in a hybridization oven (Agilent Technologies).

Microarray data analysis was done by using Limma package in R-Bioconductor. Mean foreground and median background expression values given by Agilent feature extraction Software was used for analysis. Background subtraction was done using “subtract” method in Limma, dye normalization was done by LOESS algorithm, and between array normalization was done by “quantile” method. Imfit and eBayes (Empirical Bayes method) were used to find differentially regulated genes. P value correction was done using Benjamini and Hochberg method. P value cutoff was made as 0.05. Gene enrichment analysis of the differentially regulated genes with two-fold change cut off was done using GSEA Pre Ranked Tool.

### Quantitative Real-Time PCR for microarray data validation

Total RNA from the hamster buccal pouch tissues of two individual hamsters in each experimental group was extracted using trizol reagent as described previously [48]. RNA was treated with ribonuclease free DNaseI to remove any contaminating DNA. Reverse transcription of 5 µg total RNA was performed using the oligo-dT primers. Real-time RT-PCR was performed for nine randomly chosen genes of interest as previously described using a Step One Plus thermocycler (Applied Biosystems). The PCR conditions were as follows: 95°C for 5 minutes, 40 cycles of 30 seconds at 95°C, 30 seconds at 60°C, 60 seconds at 72°C. Relative quantitative fold change compared to control was calculated using the comparative Ct method, where Ct is the cycle number at which fluorescence first exceeds the threshold. The Ct values from each sample were obtained by subtracting the values for GAPDH Ct from the target gene Ct value. Specificity of resulting PCR products was confirmed by melting curves.

### Protein extraction and Western blotting

Approximately, 50 mg of buccal pouch tissue sample from two hamsters in each experimental group was subjected to lysis in a sample buffer containing 62.5 mM Tris (pH 6.8), 2% SDS, 5% 2-mercaptoethanol, 10% glycerol and bromophenol blue. The protein concentration of lysates was determined by Bradford method. SDS-PAGE was performed using equivalent protein extracts (55 µg) from each sample. The resolved proteins were electrophoretically transferred to polyvinylidene difluoride membranes. The blots were incubated in 1× PBS containing 5% non-fat dry milk for 2 hours to block nonspecific binding sites. The blot was incubated with 1∶200 dilution of primary antibodies overnight at 4°C. After washing, the blots were incubated with 1∶1000 dilution of horseradish peroxidase-conjugated secondary antibody for 45 min at room temperature. After extensive washes with high and low salt buffers, the immunoreactive proteins were visualized using enhanced chemiluminescence detection reagents (Sigma).

### Statistical analysis

Statistical analysis was carried out using a nonparametric Mann-Whitney test (Statsdirect, United Kingdom). A probability value of less than 0.05 was considered significant.

## Supporting Information

Table S1 List of differentially expressed genes in DMBA painted animals (P = 0.05, fold change cut off- 2).
(DOC)

Table S2 List of differentially expressed genes in DMBA+chlorophyllin treated hamsters (P = 0.05, fold change cut off- 2).
(DOC)

Table S3 List of differentially expressed genes in DMBA+ellagic acid treated hamsters (P = 0.05, fold change cut off- 2).
(DOC)
