# Supplementary material for: Gene Expression Signature of DMBA-Induced Hamster Buccal Pouch Carcinomas: Modulation by Chlorophyllin and Ellagic Acid
Source: PLoS One. 2012 Apr 2;7(4):e34628. doi: 10.1371/journal.pone.0034628 (PMC3317635; doi:10.1371/journal.pone.0034628)
Supplement: Table S1 — List of differentially expressed genes in DMBA painted animals (P = 0.05, fold change cut off- 2). (DOC) [file pone.0034628.s001.doc]

| **S.No**  **Table S1.** Differentially expressed genes in DMBA painted animals (P=0.05, fold change cut off- 2). | **Systematic name** | **Gene name** | **P value** | **Fold change** |  | **S.No** | **Systematic name** | **Gene name** | **P value** | **Fold change** |
| --- | --- | --- | --- | --- | --- | --- | --- | --- | --- | --- |
|  | NM_053976 | Krt1-18 | 7.35E-10 | 4.61 |  |  | AI548388 | AI548388 | 1.12E-06 | 1.02 |
|  | S79307 | S79307 | 6.17E-10 | 4.56 |  |  | ENSRNOT00000042973 | LOC299339 | 2.37E-05 | 1.02 |
|  | NM_013059 | Alpl | 2.05E-09 | 4.42 |  |  | ENSRNOT00000026112 | Psd2_predicted | 1.34E-06 | 1.02 |
|  | X71466 | Mmp2 | 5.70E-10 | 4.15 |  |  | NM_031525 | Pdgfrb | 1.46E-06 | 1.02 |
|  | ENSRNOT00000016254 | ENSRNOT00000016254 | 2.11E-10 | 4.14 |  |  | NM_001004212 | Tusc3 | 3.11E-05 | 1.02 |
|  | ENSRNOT00000039417 | ENSRNOT00000039417 | 8.08E-11 | 3.92 |  |  | TC581743 | TC581743 | 4.57E-06 | 1.02 |
|  | ENSRNOT00000060888 | Prss22_predicted | 2.58E-09 | 3.82 |  |  | NM_012854 | Il10 | 4.56E-06 | 1.02 |
|  | ENSRNOT00000012174 | LOC499660 | 6.83E-10 | 3.82 |  |  | DV720344 | DV720344 | 1.83E-06 | 1.02 |
|  | NM_001034080 | Pyy | 1.32E-10 | 3.58 |  |  | NM_181693 | Adam28 | 3.42E-05 | 1.02 |
|  | NM_001034932 | C1qtnf6 | 8.39E-10 | 3.55 |  |  | ENSRNOT00000020609 | RGD1564549_predicted | 9.61E-07 | 1.02 |
|  | NM_031055 | Mmp9 | 4.30E-09 | 3.53 |  |  | NM_019216 | Gdf15 | 7.96E-06 | 1.02 |
|  | AA963477 | AA963477 | 3.00E-09 | 3.40 |  |  | M63970 | M63970 | 1.49E-06 | 1.02 |
|  | TC588735 | TC588735 | 9.58E-10 | 3.31 |  |  | NM_022387 | Pafah1b2 | 1.65E-06 | 1.01 |
|  | NM_031612 | Apln | 6.69E-10 | 3.16 |  |  | XM_235691 | Espl1_predicted | 2.88E-06 | 1.01 |
|  | ENSRNOT00000012658 | Sfrp2 | 1.05E-09 | 3.14 |  |  | ENSRNOT00000060750 | Sfxn1 | 7.03E-07 | 1.01 |
|  | NM_017128 | Inhba | 2.22E-09 | 3.14 |  |  | NM_001009466 | Gemin6 | 1.43E-05 | 1.01 |
|  | NM_017227 | Padi4 | 4.61E-09 | 3.11 |  |  | ENSRNOT00000020675 | ENSRNOT00000020675 | 3.02E-06 | 1.01 |
|  | NM_030845 | Cxcl1 | 6.24E-10 | 3.09 |  |  | NM_199233 | Doxl1 | 8.43E-06 | 1.01 |
|  | NM_001008750 | Ka11 | 6.48E-10 | 3.09 |  |  | CK471009 | CK471009 | 4.47E-06 | 1.01 |
|  | A_44_P944540 | A_44_P944540 | 3.95E-10 | 3.07 |  |  | NM_053664 | Sardh | 1.59E-05 | 1.01 |
|  | NM_031078 | Pde1c | 2.80E-10 | 3.07 |  |  | NM_001001718 | DERP6 | 9.54E-07 | 1.01 |
|  | NM_133311 | Il24 | 8.12E-10 | 3.02 |  |  | NM_021578 | Tgfb1 | 2.23E-06 | 1.01 |
|  | XM_344130 | Inhbb | 1.37E-09 | 3.01 |  |  | TC586229 | TC586229 | 2.16E-05 | 1.01 |
|  | BI282748 | BI282748 | 1.75E-07 | 2.92 |  |  | NM_012552 | Ela1 | 1.65E-06 | 1.01 |
|  | CB548350 | CB548350 | 2.68E-09 | 2.92 |  |  | NM_134399 | Mk1 | 2.87E-06 | 1.01 |
|  | XM_001068703 | LOC684055 | 5.98E-09 | 2.90 |  |  | NM_001008381 | Ube2f | 6.13E-07 | 1.01 |
|  | NM_177931 | Orc1l | 4.01E-09 | 2.89 |  |  | XM_217622 | RGD1560198_predicted | 2.82E-06 | 1.01 |
|  | U39609 | U39609 | 6.07E-10 | 2.86 |  |  | BC133726 | RGD1560410_predicted | 1.29E-06 | 1.01 |
|  | XM_222855 | Umpk_predicted | 5.11E-10 | 2.86 |  |  | CB544365 | CB544365 | 1.51E-06 | 1.01 |
|  | ENSRNOT00000014552 | Thbs2 | 1.02E-09 | 2.85 |  |  | NM_001024974 | RGD1311863 | 5.00E-06 | 1.01 |
|  | ENSRNOT00000002333 | RGD1306927_predicted | 9.61E-10 | 2.85 |  |  | ENSRNOT00000042056 | RGD1565459_predicted | 5.45E-06 | 1.01 |
|  | NM_212505 | Ier3 | 9.26E-09 | 2.84 |  |  | NM_031986 | Sdcbp | 1.82E-06 | 1.01 |
|  | NM_212545 | Ka17 | 2.14E-09 | 2.83 |  |  | NM_212507 | Ltb | 6.26E-07 | 1.01 |
|  | TC592522 | TC592522 | 2.89E-06 | 2.82 |  |  | ENSRNOT00000061287 | ENSRNOT00000061287 | 7.78E-05 | 1.01 |
|  | ENSRNOT00000023693 | ENSRNOT00000023693 | 1.87E-09 | 2.81 |  |  | ENSRNOT00000042005 | RGD1561543_predicted | 4.33E-06 | 1.01 |
|  | ENSRNOT00000014711 | ENSRNOT00000014711 | 2.21E-09 | 2.78 |  |  | NM_053596 | Ece1 | 2.41E-06 | 1.00 |
|  | ENSRNOT00000015712 | S100a5_predicted | 8.26E-10 | 2.78 |  |  | TC642341 | TC642341 | 0.001057 | 1.00 |
|  | NM_181478 | Rdh10 | 1.30E-08 | 2.77 |  |  | BF286666 | BF286666 | 6.50E-07 | 1.00 |
|  | XM_235417 | XM_235417 | 3.94E-09 | 2.75 |  |  | XM_219540 | RGD1310168_predicted | 8.84E-07 | 1.00 |
|  | NM_175869 | Plod2 | 1.79E-09 | 2.75 |  |  | ENSRNOT00000024276 | ENSRNOT00000024276 | 7.81E-07 | 1.00 |
|  | NM_001007265 | Os-9 | 9.27E-09 | 2.72 |  |  | AW143134 | AW143134 | 2.66E-06 | 1.00 |
|  | D63774 | Krt14 | 6.78E-10 | 2.70 |  |  | NM_053948 | Polr2g | 1.16E-05 | 1.00 |
|  | NM_212541 | Slc44a4 | 4.23E-08 | 2.69 |  |  | CA505059 | CA505059 | 3.41E-06 | 1.00 |
|  | NM_012733 | Rbp1 | 1.17E-09 | 2.69 |  |  | ENSRNOT00000042906 | ENSRNOT00000042906 | 0.001654 | 1.00 |
|  | U39608 | U39608 | 7.76E-10 | 2.68 |  |  | ENSRNOT00000032173 | RGD1305898_predicted | 2.92E-06 | 1.00 |
|  | XM_237286 | LOC301509 | 5.23E-09 | 2.67 |  |  | ENSRNOT00000042531 | ENSRNOT00000042531 | 7.39E-07 | 1.00 |
|  | NM_001024866 | RGD1311732 | 2.68E-05 | 2.67 |  |  | NM_134392 | Spata6 | 2.85E-05 | 1.00 |
|  | BF281925 | BF281925 | 2.93E-09 | 2.61 |  |  | NM_001044237 | Mcts1 | 1.88E-06 | 1.00 |
|  | BG670778 | BG670778 | 1.15E-09 | 2.61 |  |  | XM_216880 | Ndufa12_predicted | 3.08E-05 | 1.00 |
|  | NM_001000164 | Olr144_predicted | 6.85E-05 | 2.60 |  |  | NM_019205 | Ccl11 | 6.42E-06 | 1.00 |
|  | ENSRNOT00000020014 | Ccne1 | 3.34E-09 | 2.59 |  |  | ENSRNOT00000040210 | ENSRNOT00000040210 | 2.14E-06 | 1.00 |
|  | ENSRNOT00000058235 | ENSRNOT00000058235 | 2.62E-09 | 2.59 |  |  | AI044237 | AI044237 | 1.45E-05 | 1.00 |
|  | ENSRNOT00000044930 | ENSRNOT00000044930 | 4.36E-08 | 2.58 |  |  | BC092654 | Col16a1 | 3.53E-06 | 1.00 |
|  | NM_012582 | Hp | 3.44E-08 | 2.57 |  |  | ENSRNOT00000023146 | Malt1_predicted | 0.000583 | -0.99 |
|  | NM_138882 | Pspla1 | 1.67E-09 | 2.56 |  |  | XM_341948 | XM_341948 | 3.13E-05 | -0.99 |
|  | BG666662 | BG666662 | 1.04E-08 | 2.55 |  |  | NM_017088 | Gdi1 | 0.005697 | -1.00 |
|  | BQ211822 | BQ211822 | 1.25E-08 | 2.55 |  |  | XM_344998 | RGD1565190_predicted | 8.61E-05 | -1.00 |
|  | A_44_P394031 | A_44_P394031 | 1.65E-05 | 2.55 |  |  | ENSRNOT00000026646 | Eif4e2_predicted | 0.000334 | -1.00 |
|  | ENSRNOT00000005477 | Slit2 | 1.98E-08 | 2.54 |  |  | XM_001054238 | LOC680039 | 1.00E-06 | -1.01 |
|  | NM_138827 | Slc2a1 | 1.82E-09 | 2.54 |  |  | CX569439 | CX569439 | 1.02E-05 | -1.01 |
|  | NM_001034949 | LOC500573 | 1.50E-09 | 2.53 |  |  | ENSRNOT00000051257 | ENSRNOT00000051257 | 1.22E-05 | -1.01 |
|  | NM_012907 | Apobec1 | 5.38E-09 | 2.52 |  |  | NM_001009474 | Pir | 5.49E-06 | -1.01 |
|  | ENSRNOT00000028086 | Tead2 | 8.33E-09 | 2.51 |  |  | NM_031552 | Add3 | 4.18E-06 | -1.01 |
|  | ENSRNOT00000040906 | ENSRNOT00000040906 | 1.58E-07 | 2.50 |  |  | NM_019906 | Frap1 | 1.78E-05 | -1.01 |
|  | NM_022854 | Fabp9 | 6.43E-09 | 2.50 |  |  | AW915208 | AW915208 | 0.000781 | -1.01 |
|  | NM_001013994 | Rcor2 | 3.92E-09 | 2.49 |  |  | BC086535 | Gpsm1 | 1.36E-06 | -1.01 |
|  | XM_576094 | RGD1560932_predicted | 3.59E-06 | 2.48 |  |  | NM_012515 | Bzrp | 3.16E-06 | -1.01 |
|  | AA925529 | AA925529 | 2.04E-05 | 2.46 |  |  | ENSRNOT00000019707 | Vps28_predicted | 0.000129 | -1.01 |
|  | NM_021747 | Acrv1 | 8.98E-09 | 2.45 |  |  | M31178 | M31178 | 0.001899 | -1.01 |
|  | ENSRNOT00000043691 | ENSRNOT00000043691 | 7.45E-09 | 2.45 |  |  | NM_001008309 | Btf3 | 5.45E-05 | -1.01 |
|  | AI170070 | AI170070 | 8.26E-09 | 2.45 |  |  | NM_053699 | Cited4 | 1.45E-06 | -1.01 |
|  | ENSRNOT00000012219 | Rab6b_predicted | 1.80E-09 | 2.44 |  |  | NM_001033897 | RGD1309387 | 0.000137 | -1.01 |
|  | NM_019179 | Tyms | 5.38E-08 | 2.42 |  |  | BQ210985 | BQ210985 | 8.72E-05 | -1.01 |
|  | XM_574584 | RGD1566394_predicted | 8.26E-09 | 2.42 |  |  | NM_001013921 | Dtwd1 | 6.66E-07 | -1.01 |
|  | U64705 | U64705 | 2.47E-07 | 2.41 |  |  | BE102568 | BE102568 | 1.74E-06 | -1.01 |
|  | XM_343856 | Litaf | 1.31E-08 | 2.40 |  |  | NM_053895 | Frag1 | 1.48E-06 | -1.01 |
|  | NM_031327 | Cyr61 | 4.64E-08 | 2.40 |  |  | BC099150 | RGD1308009 | 0.00089 | -1.01 |
|  | U15550 | Tnc | 3.63E-07 | 2.39 |  |  | NM_134408 | Lphn2 | 7.04E-06 | -1.01 |
|  | TC591439 | TC591439 | 4.20E-09 | 2.38 |  |  | ENSRNOT00000027845 | RGD1563577_predicted | 8.80E-05 | -1.01 |
|  | ENSRNOT00000051452 | Sult5a1_predicted | 3.85E-09 | 2.38 |  |  | XR_009495 | RGD1310027_predicted | 0.000238 | -1.01 |
|  | ENSRNOT00000022469 | Loxl2_predicted | 4.49E-09 | 2.38 |  |  | ENSRNOT00000039569 | LOC310891 | 5.62E-07 | -1.01 |
|  | NM_001012198 | Arhgap9 | 6.12E-09 | 2.38 |  |  | ENSRNOT00000011619 | Mrpl15_predicted | 6.09E-05 | -1.01 |
|  | NM_001013105 | Fkbp11 | 6.83E-09 | 2.37 |  |  | ENSRNOT00000032278 | ENSRNOT00000032278 | 2.35E-05 | -1.01 |
|  | CB548031 | CB548031 | 3.25E-05 | 2.37 |  |  | TC608471 | TC608471 | 1.90E-06 | -1.01 |
|  | NM_173136 | Akr1b8 | 1.77E-08 | 2.36 |  |  | AI547958 | AI547958 | 7.78E-06 | -1.01 |
|  | NM_012676 | Tnnt2 | 5.87E-09 | 2.36 |  |  | XM_230798 | RGD1304782_predicted | 0.000473 | -1.01 |
|  | NM_017095 | Cebpe | 1.60E-07 | 2.36 |  |  | CB546740 | CB546740 | 7.91E-07 | -1.01 |
|  | XR_006746 | LOC293834 | 1.93E-09 | 2.36 |  |  | TC641555 | TC641555 | 1.06E-06 | -1.02 |
|  | NM_001008751 | Krt1-14 | 1.36E-08 | 2.36 |  |  | XM_232220 | Rybp_predicted | 6.34E-05 | -1.02 |
|  | NM_001013853 | LOC287167 | 1.11E-08 | 2.35 |  |  | ENSRNOT00000017867 | ENSRNOT00000017867 | 7.67E-06 | -1.02 |
|  | NM_053606 | Mmp23 | 4.17E-09 | 2.34 |  |  | BM382871 | BM382871 | 9.20E-07 | -1.02 |
|  | NM_001013137 | Cxcl14 | 8.21E-09 | 2.34 |  |  | ENSRNOT00000042925 | ENSRNOT00000042925 | 2.18E-05 | -1.02 |
|  | NM_019143 | Fn1 | 5.13E-08 | 2.33 |  |  | NM_012819 | Acadl | 4.34E-06 | -1.02 |
|  | CB557922 | CB557922 | 0.000326 | 2.33 |  |  | ENSRNOT00000024193 | Sema3f_predicted | 1.10E-05 | -1.02 |
|  | AY125814 | Slc1a5 | 3.63E-08 | 2.31 |  |  | NM_053306 | Pak2 | 0.000123 | -1.02 |
|  | NM_017325 | Runx1 | 1.00E-07 | 2.30 |  |  | NM_001000855 | Olr844_predicted | 5.47E-07 | -1.02 |
|  | NM_001011933 | Mlstd2 | 2.70E-07 | 2.30 |  |  | XM_341883 | Pcf11_predicted | 0.001337 | -1.02 |
|  | AW251931 | AW251931 | 5.46E-07 | 2.29 |  |  | NM_017094 | Ghr | 9.28E-06 | -1.02 |
|  | NM_022384 | Ascl1 | 5.01E-09 | 2.29 |  |  | BC083795 | LOC500855 | 4.95E-05 | -1.02 |
|  | TC638908 | TC638908 | 3.40E-09 | 2.28 |  |  | ENSRNOT00000028292 | LOC368158 | 6.49E-07 | -1.02 |
|  | NM_139185 | Gng8 | 1.00E-08 | 2.27 |  |  | A_44_P606035 | A_44_P606035 | 4.50E-05 | -1.02 |
|  | NM_053822 | S100a8 | 3.81E-09 | 2.27 |  |  | XR_006649 | LOC290902 | 4.19E-06 | -1.02 |
|  | TC575338 | TC575338 | 2.87E-09 | 2.24 |  |  | ENSRNOT00000011589 | RICS_predicted | 2.17E-05 | -1.02 |
|  | XM_346956 | RGD1564528_predicted | 2.86E-06 | 2.24 |  |  | NM_053303 | Blr1 | 7.12E-07 | -1.02 |
|  | ENSRNOT00000021954 | Cotl1_predicted | 3.55E-09 | 2.24 |  |  | ENSRNOT00000012112 | ENSRNOT00000012112 | 1.22E-06 | -1.02 |
|  | NM_017259 | Btg2 | 2.80E-08 | 2.23 |  |  | NM_021702 | Atxn3 | 1.14E-06 | -1.02 |
|  | ENSRNOT00000017833 | Syngr3_predicted | 1.31E-08 | 2.22 |  |  | BF561678 | BF561678 | 0.000209 | -1.02 |
|  | NM_177425 | Csrp2 | 7.81E-09 | 2.22 |  |  | XM_234275 | Daam1_predicted | 4.09E-07 | -1.02 |
|  | NM_053681 | S100a3 | 8.03E-07 | 2.21 |  |  | NM_001017960 | Thra | 8.00E-07 | -1.02 |
|  | ENSRNOT00000000018 | Steap1_predicted | 4.40E-08 | 2.21 |  |  | ENSRNOT00000014752 | Mrpl45_predicted | 3.14E-06 | -1.02 |
|  | AI013389 | AI013389 | 3.78E-08 | 2.20 |  |  | NM_181090 | Slc38a2 | 8.72E-07 | -1.02 |
|  | Z78279 | Col1a1 | 7.47E-09 | 2.19 |  |  | XM_001074013 | Trp53inp2 | 0.00021 | -1.03 |
|  | ENSRNOT00000003765 | Higd1b_predicted | 5.47E-09 | 2.18 |  |  | NM_053428 | Fgf13 | 1.22E-06 | -1.03 |
|  | NM_012978 | Lhcgr | 3.97E-07 | 2.18 |  |  | ENSRNOT00000048980 | Usp9x_predicted | 0.00467 | -1.03 |
|  | ENSRNOT00000005187 | ENSRNOT00000005187 | 2.74E-07 | 2.17 |  |  | ENSRNOT00000024541 | Snx2_predicted | 0.001765 | -1.03 |
|  | XM_235023 | RGD1560606_predicted | 2.74E-07 | 2.17 |  |  | NM_001047087 | Eif3s10 | 2.02E-06 | -1.03 |
|  | AA859536 | AA859536 | 3.03E-09 | 2.17 |  |  | NM_001008296 | RGD1308113 | 7.30E-06 | -1.03 |
|  | ENSRNOT00000012597 | Cdk6 | 3.62E-09 | 2.16 |  |  | ENSRNOT00000039942 | Tnks2_predicted | 3.68E-06 | -1.03 |
|  | NM_198769 | Tlr2 | 3.75E-08 | 2.15 |  |  | ENSRNOT00000008158 | Bcl11b_predicted | 0.00023 | -1.03 |
|  | XR_008037 | RGD1561455_predicted | 4.14E-09 | 2.14 |  |  | A_44_P558064 | A_44_P558064 | 5.20E-06 | -1.03 |
|  | ENSRNOT00000035926 | Arhgap22_predicted | 8.59E-09 | 2.13 |  |  | NM_001008314 | Mkrn2 | 3.07E-06 | -1.03 |
|  | ENSRNOT00000006952 | RGD1562576_predicted | 0.000212 | 2.13 |  |  | XM_343446 | Syncrip | 1.02E-06 | -1.03 |
|  | NM_030997 | Vgf | 1.37E-08 | 2.12 |  |  | NM_001004223 | Eef1g | 1.17E-06 | -1.03 |
|  | NM_001007667 | Sat | 5.37E-06 | 2.12 |  |  | U30813 | U30813 | 0.000179 | -1.03 |
|  | NM_021760 | Col5a3 | 5.50E-08 | 2.11 |  |  | ENSRNOT00000014139 | Dapp1_predicted | 7.73E-06 | -1.03 |
|  | ENSRNOT00000031175 | Crabp1 | 5.02E-08 | 2.11 |  |  | NM_001009173 | Tryx5 | 6.42E-06 | -1.03 |
|  | A_44_P767783 | A_44_P767783 | 5.28E-06 | 2.10 |  |  | XM_574316 | LOC499022 | 0.001306 | -1.03 |
|  | ENSRNOT00000005213 | ENSRNOT00000005213 | 3.64E-08 | 2.09 |  |  | ENSRNOT00000048684 | ENSRNOT00000048684 | 3.36E-06 | -1.03 |
|  | AW919536 | AW919536 | 0.000502 | 2.09 |  |  | NM_017343 | Mrlcb | 2.25E-05 | -1.03 |
|  | ENSRNOT00000049507 | RGD1560658_predicted | 3.05E-07 | 2.09 |  |  | NM_001024905 | MGC116121 | 0.000946 | -1.03 |
|  | NM_001013170 | Wars | 2.61E-08 | 2.09 |  |  | ENSRNOT00000019104 | Psmd7_predicted | 2.11E-05 | -1.03 |
|  | NM_001000963 | Olr1237_predicted | 5.14E-09 | 2.08 |  |  | NM_001012060 | Rnf146 | 1.31E-05 | -1.03 |
|  | TC622294 | TC622294 | 1.13E-07 | 2.08 |  |  | NM_012507 | Atp1b2 | 4.71E-07 | -1.03 |
|  | BE099798 | BE099798 | 1.07E-07 | 2.08 |  |  | ENSRNOT00000002634 | RGD1306226_predicted | 5.17E-06 | -1.03 |
|  | ENSRNOT00000056843 | ENSRNOT00000056843 | 2.17E-08 | 2.06 |  |  | ENSRNOT00000022875 | RGD1562529_predicted | 4.40E-06 | -1.03 |
|  | NM_031778 | Kcns3 | 1.81E-07 | 2.06 |  |  | ENSRNOT00000007809 | LOC681849 | 5.36E-06 | -1.04 |
|  | NM_001009271 | Nt5dc2 | 4.50E-08 | 2.06 |  |  | NM_178334 | Egln1 | 6.16E-05 | -1.04 |
|  | NM_024128 | Nsg1 | 2.53E-08 | 2.05 |  |  | NM_001004280 | RGD1303272 | 0.008838 | -1.04 |
|  | BF549419 | BF549419 | 2.58E-08 | 2.05 |  |  | AF010436 | AF010436 | 3.41E-05 | -1.04 |
|  | NM_017087 | Bgn | 2.08E-08 | 2.05 |  |  | NM_022402 | Arbp | 2.58E-05 | -1.04 |
|  | A_44_P419393 | A_44_P419393 | 1.66E-07 | 2.05 |  |  | NM_001007011 | Zpbp2 | 1.66E-06 | -1.04 |
|  | ENSRNOT00000001882 | Sdsl_predicted | 3.78E-05 | 2.05 |  |  | ENSRNOT00000047751 | ENSRNOT00000047751 | 4.18E-07 | -1.04 |
|  | TC585827 | TC585827 | 1.15E-08 | 2.03 |  |  | NM_001006972 | Ndufv1 | 1.24E-06 | -1.04 |
|  | NM_138828 | Apoe | 3.22E-08 | 2.03 |  |  | NM_001000997 | Olr689_predicted | 3.89E-06 | -1.04 |
|  | NM_053294 | Adora2a | 0.001729 | 2.02 |  |  | XM_221082 | RGD1559936_predicted | 7.12E-06 | -1.04 |
|  | NM_024359 | Hif1a | 9.16E-08 | 2.02 |  |  | ENSRNOT00000006325 | RGD1564811_predicted | 1.02E-06 | -1.04 |
|  | ENSRNOT00000043691 | ENSRNOT00000043691 | 4.36E-08 | 2.00 |  |  | ENSRNOT00000041114 | ENSRNOT00000041114 | 0.000366 | -1.04 |
|  | NM_207597 | Olr1078 | 9.08E-09 | 2.00 |  |  | NM_013155 | Vldlr | 0.001297 | -1.04 |
|  | ENSRNOT00000003498 | ENSRNOT00000003498 | 0.001787 | 2.00 |  |  | NM_001033684 | Cct6a | 9.87E-07 | -1.04 |
|  | A_44_P283670 | A_44_P283670 | 1.20E-07 | 1.99 |  |  | XR_006040 | LOC302804 | 0.000101 | -1.04 |
|  | ENSRNOT00000001263 | Lnx2_predicted | 2.29E-07 | 1.99 |  |  | TC631464 | TC631464 | 1.17E-06 | -1.04 |
|  | NM_201418 | Ns5atp9 | 6.62E-08 | 1.98 |  |  | NM_031352 | Dbnl | 6.71E-06 | -1.04 |
|  | BE108142 | BE108142 | 0.000727 | 1.98 |  |  | ENSRNOT00000056644 | RGD1309802_predicted | 0.004536 | -1.04 |
|  | NM_031620 | Phgdh | 1.43E-08 | 1.98 |  |  | ENSRNOT00000028174 | Rdm1_predicted | 1.66E-06 | -1.04 |
|  | ENSRNOT00000024138 | ENSRNOT00000024138 | 1.45E-08 | 1.98 |  |  | NM_001014793 | Kpna4 | 0.006186 | -1.04 |
|  | XM_001065526 | Gpr68_predicted | 3.10E-07 | 1.97 |  |  | NM_001000987 | Olr1128_predicted | 6.15E-07 | -1.04 |
|  | NM_022213 | Pik3r3 | 2.68E-05 | 1.97 |  |  | NM_001004021 | Ka13 | 1.73E-06 | -1.05 |
|  | NM_171991 | Ccnb1 | 4.07E-07 | 1.97 |  |  | NM_001025640 | RGD1305031 | 7.01E-06 | -1.05 |
|  | NM_001037336 | Lrrc4 | 1.64E-08 | 1.97 |  |  | NM_001013919 | Galk2 | 5.07E-05 | -1.05 |
|  | ENSRNOT00000013582 | Np | 9.39E-09 | 1.96 |  |  | NM_172044 | Mcpt2 | 3.60E-05 | -1.05 |
|  | AW142999 | AW142999 | 1.09E-08 | 1.96 |  |  | TC595416 | TC595416 | 8.76E-07 | -1.05 |
|  | ENSRNOT00000019335 | Myl7_predicted | 1.40E-08 | 1.96 |  |  | ENSRNOT00000039210 | ENSRNOT00000039210 | 5.19E-07 | -1.05 |
|  | TC623018 | TC623018 | 7.14E-09 | 1.96 |  |  | AF452728 | AF452728 | 1.05E-06 | -1.05 |
|  | NM_022194 | Il1rn | 1.41E-08 | 1.96 |  |  | NM_017033 | Pgm1 | 3.60E-07 | -1.05 |
|  | NM_017121 | Ctsj | 1.53E-08 | 1.96 |  |  | A_44_P314231 | A_44_P314231 | 8.10E-06 | -1.05 |
|  | ENSRNOT00000045315 | Col18a1 | 4.62E-08 | 1.95 |  |  | NM_001013192 | Olfml1 | 0.001084 | -1.05 |
|  | DV726678 | DV726678 | 2.87E-08 | 1.95 |  |  | ENSRNOT00000003066 | LOC498358 | 0.000124 | -1.05 |
|  | ENSRNOT00000058062 | RGD1560394_predicted | 7.52E-08 | 1.95 |  |  | NM_031037 | Gnb2 | 4.88E-07 | -1.05 |
|  | ENSRNOT00000000155 | Plxdc2_predicted | 4.15E-05 | 1.94 |  |  | NM_013187 | Plcg1 | 4.91E-06 | -1.05 |
|  | ENSRNOT00000020524 | Nt5c1a_predicted | 0.000233 | 1.94 |  |  | NM_001005872 | Htatip | 7.24E-06 | -1.05 |
|  | NM_019373 | Apom | 1.03E-08 | 1.93 |  |  | ENSRNOT00000047264 | RGD1566243_predicted | 0.00108 | -1.05 |
|  | TC624759 | TC624759 | 0.000143 | 1.93 |  |  | NM_001037355 | Mettl7a | 1.16E-05 | -1.05 |
|  | TC610375 | TC610375 | 7.18E-09 | 1.93 |  |  | NM_001033707 | Zfp289 | 3.58E-06 | -1.05 |
|  | ENSRNOT00000024968 | Cidea_predicted | 0.001674 | 1.93 |  |  | NM_031855 | Khk | 1.13E-06 | -1.05 |
|  | NM_001013222 | Rnd1 | 7.61E-08 | 1.93 |  |  | NM_130894 | Mfn2 | 4.27E-07 | -1.05 |
|  | ENSRNOT00000047692 | Kntc1_predicted | 1.91E-08 | 1.92 |  |  | ENSRNOT00000002684 | Ift57_predicted | 0.000254 | -1.06 |
|  | NM_173114 | Cipar1 | 3.82E-08 | 1.92 |  |  | XM_001065966 | LOC367311 | 0.002591 | -1.06 |
|  | XR_008359 | RGD1565037_predicted | 9.30E-08 | 1.91 |  |  | XM_001060221 | RGD1307395 | 2.30E-06 | -1.06 |
|  | NM_133401 | Abcb1a | 6.09E-08 | 1.91 |  |  | TC611690 | TC611690 | 0.00138 | -1.06 |
|  | ENSRNOT00000057463 | Col4a2_predicted | 2.57E-08 | 1.91 |  |  | ENSRNOT00000015573 | Tbl1xr1_predicted | 0.005583 | -1.06 |
|  | NM_001024776 | Hipk4 | 0.000105 | 1.89 |  |  | NM_139094 | Rbm16 | 0.001451 | -1.06 |
|  | ENSRNOT00000040507 | ENSRNOT00000040507 | 2.90E-07 | 1.89 |  |  | NM_175580 | Gnl3 | 4.12E-07 | -1.06 |
|  | AW913985 | AW913985 | 0.000273 | 1.89 |  |  | ENSRNOT00000028210 | Ube2q_predicted | 8.91E-06 | -1.06 |
|  | S82585 | S82585 | 1.51E-06 | 1.89 |  |  | ENSRNOT00000017268 | Spfh1_predicted | 0.000324 | -1.06 |
|  | XM_343198 | Elk3_predicted | 2.19E-08 | 1.89 |  |  | NM_022510 | Rpl4 | 0.000105 | -1.06 |
|  | CB314431 | CB314431 | 1.61E-06 | 1.89 |  |  | ENSRNOT00000043942 | ENSRNOT00000043942 | 0.000246 | -1.06 |
|  | NM_001009679 | Psph | 9.82E-09 | 1.89 |  |  | NM_001025639 | LOC292543 | 1.63E-06 | -1.06 |
|  | ENSRNOT00000026375 | Zmynd15_predicted | 1.33E-08 | 1.88 |  |  | ENSRNOT00000045519 | Dock9 | 0.000101 | -1.06 |
|  | NM_012889 | Vcam1 | 2.35E-08 | 1.87 |  |  | AY280961 | Tmem23 | 2.61E-06 | -1.06 |
|  | NM_031771 | Thbd | 1.05E-06 | 1.87 |  |  | TC642306 | TC642306 | 1.73E-06 | -1.06 |
|  | NM_001004274 | Igfbp4 | 2.82E-08 | 1.87 |  |  | NM_001025625 | Stub1 | 1.15E-05 | -1.06 |
|  | XM_222476 | Rtbnd_predicted | 1.10E-05 | 1.87 |  |  | ENSRNOT00000021637 | RGD1562691_predicted | 4.98E-06 | -1.06 |
|  | BE111887 | BE111887 | 0.00071 | 1.86 |  |  | NM_053880 | Dncic2 | 4.07E-05 | -1.06 |
|  | DV728037 | DV728037 | 1.52E-08 | 1.85 |  |  | ENSRNOT00000009318 | Cep57 | 0.004838 | -1.07 |
|  | AI409745 | AI409745 | 4.36E-08 | 1.85 |  |  | NM_001037355 | Mettl7a | 3.51E-06 | -1.07 |
|  | A_44_P985259 | A_44_P985259 | 0.000138 | 1.84 |  |  | ENSRNOT00000060784 | RGD1566272_predicted | 7.81E-06 | -1.07 |
|  | NM_022635 | Cml4 | 2.91E-08 | 1.84 |  |  | BC087069 | BC087069 | 1.14E-06 | -1.07 |
|  | XM_214014 | XM_214014 | 9.95E-09 | 1.84 |  |  | ENSRNOT00000017483 | Dupd1_predicted | 5.44E-07 | -1.07 |
|  | ENSRNOT00000027972 | Phlda2_predicted | 1.18E-07 | 1.84 |  |  | ENSRNOT00000008703 | RGD1560139_predicted | 3.58E-07 | -1.07 |
|  | BI297059 | BI297059 | 7.65E-06 | 1.84 |  |  | NM_001024339 | LOC500377 | 3.75E-07 | -1.07 |
|  | NR_001567 | Terc | 4.80E-08 | 1.84 |  |  | AI175258 | AI175258 | 9.95E-07 | -1.07 |
|  | XM_001076829 | LOC687039 | 4.13E-08 | 1.84 |  |  | XR_005646 | LOC364806 | 0.000196 | -1.07 |
|  | ENSRNOT00000017339 | RGD1564316_predicted | 1.01E-07 | 1.84 |  |  | NM_017236 | Pebp1 | 2.31E-06 | -1.07 |
|  | NM_012823 | Anxa3 | 6.41E-05 | 1.84 |  |  | NM_031805 | Ank3 | 6.34E-07 | -1.07 |
|  | NM_021261 | Tmsb10 | 6.92E-07 | 1.83 |  |  | BF289687 | BF289687 | 2.17E-06 | -1.07 |
|  | ENSRNOT00000038863 | RGD1304731_predicted | 3.57E-07 | 1.83 |  |  | AI705009 | AI705009 | 3.63E-07 | -1.07 |
|  | ENSRNOT00000016519 | S100a13_predicted | 2.51E-08 | 1.83 |  |  | AW142013 | AW142013 | 8.82E-07 | -1.07 |
|  | ENSRNOT00000045463 | LOC680591 | 1.10E-07 | 1.83 |  |  | NM_019131 | Tpm1 | 3.89E-07 | -1.07 |
|  | A_44_P745478 | A_44_P745478 | 1.98E-08 | 1.83 |  |  | DV727231 | DV727231 | 0.000454 | -1.07 |
|  | BI283491 | BI283491 | 6.43E-08 | 1.83 |  |  | ENSRNOT00000043709 | ENSRNOT00000043709 | 7.83E-07 | -1.07 |
|  | ENSRNOT00000016164 | ENSRNOT00000016164 | 4.96E-08 | 1.82 |  |  | NM_080581 | Abcc3 | 3.18E-05 | -1.07 |
|  | BP503690 | BP503690 | 1.27E-08 | 1.82 |  |  | ENSRNOT00000000464 | Gopc_predicted | 4.53E-05 | -1.07 |
|  | XR_009008 | RGD1307390_predicted | 3.17E-07 | 1.81 |  |  | ENSRNOT00000028262 | ENSRNOT00000028262 | 6.47E-07 | -1.07 |
|  | NM_013079 | Asns | 1.91E-08 | 1.81 |  |  | XM_233699 | XM_233699 | 4.37E-07 | -1.08 |
|  | ENSRNOT00000005073 | Col5a2 | 6.91E-07 | 1.81 |  |  | NM_145084 | Retsat | 4.15E-06 | -1.08 |
|  | NM_031148 | Slc20a1 | 6.30E-08 | 1.81 |  |  | XM_217278 | XM_217278 | 3.39E-05 | -1.08 |
|  | NM_053949 | Kcnh2 | 1.08E-08 | 1.81 |  |  | ENSRNOT00000025574 | LOC293589 | 9.48E-05 | -1.08 |
|  | NM_012699 | Dnajb9 | 1.84E-05 | 1.81 |  |  | ENSRNOT00000006696 | ENSRNOT00000006696 | 5.11E-07 | -1.08 |
|  | BQ199904 | BQ199904 | 7.14E-06 | 1.80 |  |  | NM_001024757 | Wwp1 | 0.000162 | -1.08 |
|  | NM_053317 | Nr0b1 | 1.63E-08 | 1.80 |  |  | NM_138843 | Mpst | 2.88E-07 | -1.08 |
|  | NM_031005 | Actn1 | 8.63E-06 | 1.80 |  |  | NM_001024332 | LOC500282 | 0.001684 | -1.08 |
|  | ENSRNOT00000020323 | Ube2c_predicted | 1.78E-08 | 1.80 |  |  | ENSRNOT00000051317 | Hspb7 | 1.25E-06 | -1.08 |
|  | NM_001013960 | LOC302640 | 1.15E-07 | 1.79 |  |  | ENSRNOT00000047053 | Srebf1 | 7.03E-07 | -1.08 |
|  | ENSRNOT00000007761 | RGD1306498_predicted | 2.27E-08 | 1.79 |  |  | NM_001034829 | RGD1309471 | 5.36E-05 | -1.08 |
|  | NM_031736 | Slc27a2 | 2.13E-05 | 1.79 |  |  | NM_001015010 | Cib2 | 2.56E-06 | -1.08 |
|  | AI574843 | AI574843 | 1.58E-05 | 1.78 |  |  | NM_033235 | Mdh1 | 8.66E-06 | -1.08 |
|  | NM_001033064 | Kazald1 | 1.65E-05 | 1.78 |  |  | NM_031624 | Igbp1 | 3.62E-06 | -1.09 |
|  | XM_219680 | RGD1306343_predicted | 1.69E-07 | 1.78 |  |  | NM_022382 | Pde4dip | 9.85E-06 | -1.09 |
|  | NM_001039505 | Irx2 | 2.15E-08 | 1.78 |  |  | NM_019332 | Padi1 | 6.46E-07 | -1.09 |
|  | NM_012715 | Adm | 5.53E-08 | 1.78 |  |  | TC592843 | TC592843 | 0.002277 | -1.09 |
|  | NM_022195 | Ivl | 1.84E-08 | 1.78 |  |  | A_44_P215488 | A_44_P215488 | 0.003621 | -1.09 |
|  | A_44_P728923 | A_44_P728923 | 3.06E-07 | 1.77 |  |  | NM_001013139 | Fars2 | 1.21E-05 | -1.09 |
|  | AI013718 | AI013718 | 3.87E-07 | 1.77 |  |  | ENSRNOT00000001871 | LOC304361 | 3.06E-05 | -1.09 |
|  | BE119393 | BE119393 | 9.92E-05 | 1.77 |  |  | TC601834 | TC601834 | 1.01E-05 | -1.09 |
|  | XM_001054985 | LOC679924 | 2.10E-05 | 1.77 |  |  | NM_019152 | Capn1 | 1.94E-06 | -1.09 |
|  | XM_240367 | RGD1563825_predicted | 0.000141 | 1.76 |  |  | XM_574414 | LOC499120 | 8.37E-05 | -1.09 |
|  | NM_022205 | Cxcr4 | 2.30E-06 | 1.76 |  |  | XR_006664 | LOC499224 | 1.53E-05 | -1.09 |
|  | NM_053846 | Nrxn2 | 1.20E-07 | 1.76 |  |  | ENSRNOT00000003707 | Dhx9_predicted | 0.000354 | -1.09 |
|  | TC605360 | TC605360 | 0.000242 | 1.76 |  |  | TC614891 | TC614891 | 8.20E-07 | -1.09 |
|  | TC617303 | TC617303 | 3.66E-08 | 1.76 |  |  | ENSRNOT00000015628 | Mocs1_predicted | 3.17E-07 | -1.09 |
|  | BU946660 | Sftpc | 6.13E-06 | 1.75 |  |  | ENSRNOT00000004097 | RGD1311526_predicted | 1.04E-05 | -1.10 |
|  | NM_001008280 | Lrrc59 | 6.03E-08 | 1.75 |  |  | XM_213991 | XM_213991 | 1.41E-06 | -1.10 |
|  | NM_019361 | Arc | 2.09E-08 | 1.75 |  |  | XM_001060522 | RGD1562533_predicted | 1.77E-06 | -1.10 |
|  | BF555731 | BF555731 | 2.19E-08 | 1.75 |  |  | BE117215 | BE117215 | 3.97E-07 | -1.10 |
|  | BF522334 | BF522334 | 1.26E-07 | 1.75 |  |  | NM_031091 | Rab3b | 3.28E-07 | -1.10 |
|  | NM_001047101 | Adamts7_predicted | 2.28E-08 | 1.74 |  |  | NM_053776 | Dnajc2 | 5.82E-06 | -1.10 |
|  | AI236332 | AI236332 | 6.32E-08 | 1.74 |  |  | NM_053456 | Plcl1 | 1.24E-06 | -1.10 |
|  | ENSRNOT00000058611 | RGD1305797_predicted | 0.000302 | 1.74 |  |  | DV716406 | DV716406 | 1.87E-06 | -1.10 |
|  | NM_053647 | Cxcl2 | 2.45E-08 | 1.73 |  |  | NM_138976 | Mfn1 | 2.05E-06 | -1.10 |
|  | XM_001055813 | LOC680122 | 2.91E-08 | 1.73 |  |  | ENSRNOT00000039540 | Ppfia4 | 3.38E-07 | -1.10 |
|  | NM_031345 | Tsc22d3 | 3.75E-08 | 1.73 |  |  | NM_030987 | Gnb1 | 1.81E-05 | -1.10 |
|  | ENSRNOT00000035459 | Hbq1_predicted | 3.99E-08 | 1.72 |  |  | XM_213336 | Aloxe3_predicted | 3.10E-06 | -1.10 |
|  | TC598512 | TC598512 | 2.32E-08 | 1.72 |  |  | NM_031561 | Cd36 | 3.30E-05 | -1.10 |
|  | NM_001097581 | Sav1_predicted | 4.71E-08 | 1.71 |  |  | ENSRNOT00000042377 | Sort1 | 2.40E-06 | -1.10 |
|  | NM_001029916 | RGD1309720 | 1.38E-07 | 1.7 |  |  | ENSRNOT00000040029 | ENSRNOT00000040029 | 4.83E-07 | -1.11 |
|  | NM_017320 | Ctss | 5.13E-08 | 1.71 |  |  | AI602844 | AI602844 | 6.58E-07 | -1.11 |
|  | NM_024151 | Arf4 | 7.76E-08 | 1.70 |  |  | NM_053631 | Banf1 | 0.000447 | -1.11 |
|  | ENSRNOT00000012985 | Cdkn3_predicted | 2.50E-08 | 1.70 |  |  | ENSRNOT00000058872 | Gtf2e2_predicted | 0.004158 | -1.11 |
|  | NM_053633 | Egr2 | 4.34E-07 | 1.70 |  |  | CX570116 | CX570116 | 3.82E-07 | -1.11 |
|  | AI407755 | AI407755 | 1.33E-07 | 1.69 |  |  | NM_057186 | Hadhsc | 1.08E-05 | -1.11 |
|  | ENSRNOT00000020260 | Il18rap | 4.66E-06 | 1.69 |  |  | ENSRNOT00000008892 | Setmar | 0.000169 | -1.11 |
|  | ENSRNOT00000031679 | RGD1561402_predicted | 4.26E-08 | 1.69 |  |  | NM_207591 | Gltscr2 | 3.12E-07 | -1.11 |
|  | ENSRNOT00000043831 | ttc21a_predicted | 1.38E-07 | 1.69 |  |  | BG373817 | BG373817 | 2.52E-07 | -1.11 |
|  | ENSRNOT00000026873 | Rbm35b_predicted | 7.60E-08 | 1.68 |  |  | XM_228999 | XM_228999 | 1.62E-06 | -1.11 |
|  | NM_001024983 | RGD1559532_predicted | 5.21E-07 | 1.68 |  |  | CB546110 | CB546110 | 3.09E-05 | -1.11 |
|  | XM_340963 | RGD1563888_predicted | 8.99E-08 | 1.68 |  |  | ENSRNOT00000005385 | Snx12_predicted | 0.000155 | -1.11 |
|  | NM_213567 | Znrd1 | 2.52E-08 | 1.68 |  |  | TC614770 | TC614770 | 6.75E-07 | -1.11 |
|  | XM_001077010 | LOC691149 | 1.95E-08 | 1.68 |  |  | ENSRNOT00000021857 | RGD1562968_predicted | 9.12E-07 | -1.11 |
|  | AW525315 | AW525315 | 0.000643 | 1.68 |  |  | NM_145678 | Vps4a | 1.25E-06 | -1.12 |
|  | TC621252 | TC621252 | 2.94E-08 | 1.68 |  |  | NM_024392 | Hsd17b4 | 6.76E-07 | -1.12 |
|  | TC634337 | TC634337 | 3.20E-08 | 1.68 |  |  | ENSRNOT00000054976 | ENSRNOT00000054976 | 4.39E-07 | -1.12 |
|  | BI275716 | BI275716 | 6.51E-08 | 1.68 |  |  | NM_013177 | Got2 | 1.43E-06 | -1.12 |
|  | NM_012620 | Serpine1 | 2.46E-08 | 1.67 |  |  | XM_342171 | RGD1562968_predicted | 7.12E-07 | -1.12 |
|  | ENSRNOT00000002156 | Evx2_predicted | 5.88E-05 | 1.67 |  |  | NM_001044394 | Ablim1_predicted | 4.99E-06 | -1.12 |
|  | ENSRNOT00000032645 | Nalp5_predicted | 1.04E-07 | 1.66 |  |  | ENSRNOT00000010986 | RGD1563028_predicted | 6.22E-07 | -1.12 |
|  | NM_134458 | Psmc3ip | 1.57E-07 | 1.66 |  |  | NM_024398 | Aco2 | 2.85E-06 | -1.12 |
|  | ENSRNOT00000016430 | ENSRNOT00000016430 | 5.40E-07 | 1.65 |  |  | TC618580 | TC618580 | 0.000116 | -1.12 |
|  | XM_213845 | RGD1307753_predicted | 3.93E-07 | 1.65 |  |  | NM_032615 | Mir16 | 6.15E-07 | -1.12 |
|  | NM_031507 | Egfr | 2.76E-07 | 1.64 |  |  | TC594408 | TC594408 | 3.32E-06 | -1.12 |
|  | NM_053968 | Mt3 | 4.31E-08 | 1.64 |  |  | NM_031648 | Fxyd1 | 5.32E-06 | -1.12 |
|  | ENSRNOT00000057140 | ENSRNOT00000057140 | 8.45E-08 | 1.64 |  |  | NM_017184 | Tnni1 | 2.88E-05 | -1.12 |
|  | CK843267 | CK843267 | 1.29E-06 | 1.63 |  |  | ENSRNOT00000009998 | LOC686184 | 4.83E-06 | -1.12 |
|  | TC591551 | TC591551 | 4.50E-08 | 1.63 |  |  | AA799593 | AA799593 | 4.98E-07 | -1.13 |
|  | AA800053 | AA800053 | 1.63E-06 | 1.63 |  |  | NM_001024275 | Rassf4 | 4.34E-07 | -1.13 |
|  | AW914942 | AW914942 | 2.69E-07 | 1.63 |  |  | ENSRNOT00000019126 | Sf3b1 | 0.007089 | -1.13 |
|  | NM_172047 | Eaf2 | 4.66E-08 | 1.63 |  |  | ENSRNOT00000007927 | Rpia_predicted | 6.10E-05 | -1.13 |
|  | NM_001034104 | Tmc4 | 3.40E-07 | 1.62 |  |  | NM_001014246 | RGD1309482 | 6.49E-07 | -1.13 |
|  | NM_001025749 | Grap | 1.44E-07 | 1.62 |  |  | ENSRNOT00000009816 | ENSRNOT00000009816 | 4.77E-06 | -1.13 |
|  | CA506147 | CA506147 | 6.56E-07 | 1.62 |  |  | ENSRNOT00000007073 | Smc6l1_predicted | 0.001034 | -1.13 |
|  | A_44_P470717 | A_44_P470717 | 2.45E-07 | 1.62 |  |  | U78139 | Zfp637 | 0.003326 | -1.13 |
|  | AF348365 | AF348365 | 3.65E-08 | 1.61 |  |  | NM_133583 | Ndrg2 | 1.10E-06 | -1.13 |
|  | ENSRNOT00000016357 | ENSRNOT00000016357 | 6.24E-06 | 1.61 |  |  | A_44_P504061 | A_44_P504061 | 3.24E-07 | -1.13 |
|  | NM_001004259 | Pnkp | 5.92E-08 | 1.61 |  |  | ENSRNOT00000046486 | Tiam1 | 2.25E-06 | -1.13 |
|  | XM_219128 | Dchs1_predicted | 1.05E-07 | 1.61 |  |  | ENSRNOT00000038521 | Zfp637 | 0.001024 | -1.13 |
|  | BI282039 | BI282039 | 0.000128 | 1.60 |  |  | TC599165 | TC599165 | 4.18E-07 | -1.13 |
|  | BI290979 | BI290979 | 6.79E-05 | 1.60 |  |  | ENSRNOT00000032301 | RGD1310185_predicted | 0.002352 | -1.13 |
|  | NM_031337 | St3gal5 | 8.11E-07 | 1.60 |  |  | XR_006475 | LOC499479 | 0.000677 | -1.13 |
|  | ENSRNOT00000019574 | Txnl5_predicted | 6.21E-08 | 1.60 |  |  | NM_133429 | Zfp384 | 7.06E-05 | -1.14 |
|  | AA997505 | AA997505 | 1.53E-06 | 1.59 |  |  | XR_006708 | LOC291871 | 2.62E-05 | -1.14 |
|  | AI548404 | AI548404 | 2.09E-05 | 1.59 |  |  | NM_172331 | Vps24 | 7.92E-06 | -1.14 |
|  | NM_001039196 | Slc39a13 | 6.66E-08 | 1.59 |  |  | NM_001024314 | LOC499913 | 3.52E-07 | -1.14 |
|  | ENSRNOT00000006402 | ENSRNOT00000006402 | 4.28E-08 | 1.58 |  |  | NM_181087 | Cyp26b1 | 6.47E-06 | -1.14 |
|  | ENSRNOT00000006454 | Flrt3_predicted | 1.01E-06 | 1.58 |  |  | NM_013015 | Ptgds | 2.84E-07 | -1.14 |
|  | ENSRNOT00000044611 | Spbc24_predicted | 8.93E-08 | 1.57 |  |  | NM_053365 | Fabp4 | 0.001479 | -1.14 |
|  | XM_573948 | Irs2 | 3.93E-07 | 1.57 |  |  | NM_001014184 | LOC362261 | 6.63E-07 | -1.15 |
|  | AI639318 | AI639318 | 0.000293 | 1.57 |  |  | NM_153469 | Pkig | 2.61E-06 | -1.15 |
|  | AI549033 | AI549033 | 6.38E-08 | 1.57 |  |  | NM_001012039 | Efemp1 | 3.43E-05 | -1.15 |
|  | XM_230870 | RGD1561993_predicted | 1.34E-07 | 1.57 |  |  | NM_012686 | Vsnl1 | 5.26E-05 | -1.15 |
|  | XR_006448 | LOC498601 | 6.51E-08 | 1.57 |  |  | ENSRNOT00000019025 | ENSRNOT00000019025 | 1.52E-06 | -1.15 |
|  | ENSRNOT00000056554 | Ly6h_predicted | 1.69E-06 | 1.57 |  |  | NM_001033757 | Cdkn1c | 6.80E-07 | -1.15 |
|  | NM_139254 | Tubb3 | 4.32E-08 | 1.56 |  |  | ENSRNOT00000059191 | ENSRNOT00000059191 | 1.71E-06 | -1.15 |
|  | XM_215666 | Trim45_predicted | 4.11E-07 | 1.56 |  |  | DV729262 | DV729262 | 2.76E-07 | -1.15 |
|  | NM_153727 | Gpr3 | 9.31E-08 | 1.56 |  |  | A_44_P958935 | A_44_P958935 | 2.92E-06 | -1.15 |
|  | NM_012690 | Abcb4 | 1.77E-06 | 1.56 |  |  | NM_001008281 | Psmd3 | 4.06E-06 | -1.15 |
|  | A_44_P185402 | A_44_P185402 | 3.69E-08 | 1.55 |  |  | NM_177933 | Sel1h | 4.94E-07 | -1.15 |
|  | NM_033099 | Ptprv | 8.54E-08 | 1.55 |  |  | NM_198753 | Rpl3 | 0.000183 | -1.15 |
|  | BC062235 | Hnrpa1 | 4.82E-06 | 1.55 |  |  | TC595980 | TC595980 | 1.97E-07 | -1.15 |
|  | ENSRNOT00000050055 | Ltk_predicted | 2.23E-07 | 1.55 |  |  | ENSRNOT00000017233 | RGD1560011_predicted | 1.62E-06 | -1.15 |
|  | AA925160 | AA925160 | 4.05E-06 | 1.55 |  |  | NM_017115 | Myog | 1.86E-06 | -1.15 |
|  | NM_134452 | Col5a1 | 2.90E-07 | 1.55 |  |  | NM_001004132 | Pctk1 | 1.98E-06 | -1.15 |
|  | XM_343173 | Syde1_predicted | 8.49E-08 | 1.55 |  |  | NM_017240 | Myh7 | 5.18E-07 | -1.16 |
|  | NM_030826 | Gpx1 | 5.05E-08 | 1.55 |  |  | ENSRNOT00000048026 | Rnasen | 0.002455 | -1.16 |
|  | NM_021653 | Dio1 | 1.35E-07 | 1.54 |  |  | ENSRNOT00000019642 | Psmd13_predicted | 0.000206 | -1.16 |
|  | ENSRNOT00000039905 | ENSRNOT00000039905 | 1.76E-07 | 1.54 |  |  | TC618498 | TC618498 | 2.91E-07 | -1.16 |
|  | ENSRNOT00000055987 | ENSRNOT00000055987 | 4.57E-06 | 1.54 |  |  | CR754090 | CR754090 | 2.45E-07 | -1.16 |
|  | A_44_P613180 | A_44_P613180 | 9.85E-06 | 1.54 |  |  | XM_343421 | XM_343421 | 1.04E-06 | -1.16 |
|  | XM_216966 | Slurp1_predicted | 8.96E-08 | 1.53 |  |  | ENSRNOT00000002692 | Zbtb20_predicted | 1.16E-06 | -1.16 |
|  | NM_031514 | Jak2 | 4.58E-07 | 1.53 |  |  | NM_001014157 | Ltv1 | 0.000986 | -1.16 |
|  | ENSRNOT00000051095 | ENSRNOT00000051095 | 0.000139 | 1.53 |  |  | XR_006893 | LOC288019 | 4.44E-05 | -1.16 |
|  | NM_133425 | Ppp1r14c | 8.05E-07 | 1.53 |  |  | XM_223012 | Trp53bp2_predicted | 7.45E-07 | -1.16 |
|  | XM_343253 | RGD1305976_predicted | 1.41E-07 | 1.53 |  |  | NM_012489 | Acaa1 | 4.48E-07 | -1.16 |
|  | AW916210 | AW916210 | 1.26E-07 | 1.53 |  |  | NM_001037645 | Rab2b | 6.13E-06 | -1.16 |
|  | AI231472 | AI231472 | 5.62E-07 | 1.53 |  |  | NM_001013126 | Cyb5r1 | 1.79E-05 | -1.17 |
|  | BF562116 | BF562116 | 1.08E-05 | 1.53 |  |  | ENSRNOT00000010682 | LOC362477 | 0.000501 | -1.17 |
|  | BF285467 | BF285467 | 2.08E-07 | 1.52 |  |  | NM_001011926 | Fts | 0.000223 | -1.17 |
|  | NM_017159 | Hal | 7.31E-08 | 1.52 |  |  | NM_020088 | Odz2 | 2.44E-07 | -1.17 |
|  | ENSRNOT00000037023 | RGD1562705_predicted | 3.22E-07 | 1.52 |  |  | A_44_P485409 | A_44_P485409 | 8.61E-06 | -1.17 |
|  | NM_022293 | Kcnk13 | 6.84E-06 | 1.52 |  |  | AI137604 | AI137604 | 2.98E-07 | -1.17 |
|  | NM_001006997 | Smpd1 | 4.46E-08 | 1.52 |  |  | ENSRNOT00000024667 | Slc23a3_predicted | 2.40E-07 | -1.17 |
|  | BF564017 | BF564017 | 7.21E-08 | 1.52 |  |  | NM_053698 | Cited2 | 4.68E-07 | -1.17 |
|  | AW913892 | AW913892 | 4.14E-08 | 1.52 |  |  | NM_001014196 | RGD1309207 | 0.003247 | -1.17 |
|  | TC604480 | TC604480 | 6.10E-07 | 1.52 |  |  | ENSRNOT00000000078 | Ppp2r5a_predicted | 7.06E-07 | -1.17 |
|  | TC631080 | TC631080 | 2.74E-05 | 1.52 |  |  | AW914767 | AW914767 | 3.10E-07 | -1.17 |
|  | XM_343200 | Plxnc1_predicted | 1.98E-07 | 1.52 |  |  | NM_013132 | Anxa5 | 3.32E-05 | -1.17 |
|  | NM_053370 | Timm8a | 4.69E-08 | 1.51 |  |  | NM_001001514 | Ablim2 | 3.58E-07 | -1.17 |
|  | ENSRNOT00000046054 | Bmp1 | 4.61E-08 | 1.51 |  |  | AW143016 | AW143016 | 6.19E-07 | -1.17 |
|  | ENSRNOT00000011383 | RGD1559463_predicted | 7.80E-07 | 1.51 |  |  | NM_138919 | Unc50 | 0.003986 | -1.17 |
|  | ENSRNOT00000010210 | Cse1l_predicted | 0.00177 | 1.51 |  |  | XM_346066 | XM_346066 | 5.70E-05 | -1.17 |
|  | ENSRNOT00000056736 | Ep400 | 2.13E-07 | 1.51 |  |  | XM_213799 | RGD1562579_predicted | 4.63E-07 | -1.18 |
|  | ENSRNOT00000017217 | Col15a1 | 1.57E-07 | 1.50 |  |  | CB546969 | CB546969 | 0.006962 | -1.18 |
|  | A_44_P347431 | A_44_P347431 | 1.33E-05 | 1.50 |  |  | XM_001065707 | RGD1566031_predicted | 4.90E-06 | -1.18 |
|  | NM_012927 | Cdh6 | 4.84E-07 | 1.50 |  |  | NM_001025010 | RGD1311293 | 1.53E-07 | -1.18 |
|  | NM_020102 | Mos | 3.95E-07 | 1.50 |  |  | NM_001014161 | LOC361596 | 1.09E-06 | -1.18 |
|  | NM_173137 | Fads3 | 1.32E-07 | 1.50 |  |  | NM_172023 | Osbpl1a | 3.49E-06 | -1.18 |
|  | BM385724 | BM385724 | 6.06E-08 | 1.50 |  |  | BF523141 | BF523141 | 2.46E-06 | -1.18 |
|  | AW917031 | AW917031 | 1.25E-07 | 1.50 |  |  | ENSRNOT00000033858 | Lrrc28_predicted | 2.30E-06 | -1.18 |
|  | ENSRNOT00000047475 | Bub1_predicted | 1.97E-07 | 1.50 |  |  | NM_001044279 | LOC681694 | 2.67E-07 | -1.18 |
|  | NM_199091 | Cct3 | 8.86E-08 | 1.50 |  |  | BF286299 | BF286299 | 9.51E-07 | -1.18 |
|  | NM_019237 | Pcolce | 3.63E-07 | 1.49 |  |  | ENSRNOT00000008630 | Fyco1_predicted | 7.30E-07 | -1.18 |
|  | ENSRNOT00000011123 | Rpa3_predicted | 5.07E-08 | 1.49 |  |  | ENSRNOT00000038125 | RGD1305774_predicted | 8.73E-07 | -1.18 |
|  | NM_031318 | Tctex1 | 3.40E-07 | 1.49 |  |  | NM_019196 | Mpdz | 4.11E-06 | -1.18 |
|  | XM_001063400 | RGD1563510_predicted | 6.40E-08 | 1.49 |  |  | ENSRNOT00000009008 | Sumf1_predicted | 0.000453 | -1.18 |
|  | BE119385 | BE119385 | 0.002244 | 1.48 |  |  | BC061963 | MGC72627 | 1.91E-07 | -1.18 |
|  | BC092654 | Col16a1 | 1.23E-07 | 1.48 |  |  | NM_001014190 | RGD1307218 | 2.34E-06 | -1.18 |
|  | NM_031841 | Scd2 | 4.86E-07 | 1.48 |  |  | TC580778 | TC580778 | 0.014688 | -1.18 |
|  | NM_024388 | Nr4a1 | 1.18E-07 | 1.48 |  |  | A_44_P965923 | A_44_P965923 | 4.71E-06 | -1.19 |
|  | ENSRNOT00000011138 | ENSRNOT00000011138 | 8.03E-07 | 1.47 |  |  | NM_053459 | Rab27b | 5.84E-07 | -1.19 |
|  | NM_181086 | Tnfrsf12a | 1.39E-06 | 1.47 |  |  | NM_031235 | Pard3 | 2.59E-06 | -1.19 |
|  | NM_138850 | Fap | 2.61E-06 | 1.47 |  |  | NM_057152 | Keap1 | 5.37E-07 | -1.19 |
|  | TC619875 | TC619875 | 5.95E-07 | 1.47 |  |  | AW142402 | AW142402 | 0.000233 | -1.19 |
|  | NM_012643 | Ret | 7.05E-08 | 1.47 |  |  | ENSRNOT00000006903 | Ccdc53_predicted | 0.001156 | -1.19 |
|  | NM_001025049 | Acrbp | 2.10E-07 | 1.47 |  |  | ENSRNOT00000022117 | Galnt1 | 0.000145 | -1.19 |
|  | NM_133537 | Expi | 2.08E-07 | 1.47 |  |  | BF281819 | BF281819 | 2.74E-06 | -1.19 |
|  | U18650 | Hdh | 4.26E-05 | 1.47 |  |  | NM_001008289 | Sbds | 5.06E-06 | -1.19 |
|  | TC625597 | TC625597 | 2.60E-06 | 1.46 |  |  | NM_138837 | Pou3f3 | 6.29E-07 | -1.19 |
|  | XM_235679 | XM_235679 | 3.44E-07 | 1.46 |  |  | ENSRNOT00000013353 | Siahbp1 | 0.000105 | -1.19 |
|  | BQ196729 | S100a6 | 1.19E-07 | 1.46 |  |  | NM_024155 | Anxa4 | 0.000118 | -1.19 |
|  | ENSRNOT00000025653 | RGD1565584_predicted | 1.76E-05 | 1.46 |  |  | ENSRNOT00000023588 | Tscot_predicted | 3.78E-07 | -1.19 |
|  | ENSRNOT00000056074 | ENSRNOT00000056074 | 8.40E-08 | 1.46 |  |  | NM_001024754 | Gmps | 0.00975 | -1.20 |
|  | NM_012904 | Anxa1 | 7.15E-08 | 1.46 |  |  | TC586471 | TC586471 | 0.001042 | -1.20 |
|  | NM_001025401 | Pcnt1 | 2.84E-07 | 1.45 |  |  | AA926202 | AA926202 | 8.81E-06 | -1.20 |
|  | ENSRNOT00000045454 | RGD1564947_predicted | 4.29E-07 | 1.45 |  |  | NM_001014028 | RGD1305246 | 0.000289 | -1.20 |
|  | XM_001073977 | RGD1566130_predicted | 2.93E-06 | 1.45 |  |  | NM_053549 | Vegfb | 2.53E-06 | -1.20 |
|  | NM_053768 | Uox | 1.12E-05 | 1.45 |  |  | EF076766 | EF076766 | 0.000441 | -1.20 |
|  | CK842666 | CK842666 | 1.11E-07 | 1.45 |  |  | NM_001000664 | Olr569_predicted | 4.41E-06 | -1.20 |
|  | NM_013066 | Mtap2 | 3.16E-07 | 1.45 |  |  | AF327071 | Arntl2 | 2.67E-07 | -1.20 |
|  | AA965084 | AA965084 | 2.69E-07 | 1.44 |  |  | XM_240311 | Clybl | 1.07E-06 | -1.20 |
|  | ENSRNOT00000036203 | Nanos1_predicted | 1.11E-06 | 1.44 |  |  | AI072072 | AI072072 | 1.31E-06 | -1.20 |
|  | NM_022686 | Hist1h4b | 0.000102 | 1.44 |  |  | XM_001062857 | Cnot6l_predicted | 3.89E-05 | -1.20 |
|  | XM_233037 | Pappa_predicted | 3.12E-07 | 1.44 |  |  | NM_057147 | Sec22l2 | 0.000922 | -1.20 |
|  | NM_001012125 | Loxl1 | 1.67E-07 | 1.44 |  |  | A_44_P667548 | A_44_P667548 | 0.000111 | -1.20 |
|  | ENSRNOT00000007351 | ENSRNOT00000007351 | 8.46E-07 | 1.44 |  |  | NM_001013136 | Rfxank | 5.66E-06 | -1.20 |
|  | XM_216872 | Nup37_predicted | 1.41E-07 | 1.44 |  |  | NM_031795 | Ugcg | 8.80E-07 | -1.20 |
|  | NM_012588 | Igfbp3 | 5.75E-06 | 1.43 |  |  | ENSRNOT00000044127 | ENSRNOT00000044127 | 5.57E-07 | -1.20 |
|  | ENSRNOT00000018964 | Rnf151_predicted | 4.51E-06 | 1.43 |  |  | NM_017112 | Hpn | 1.70E-07 | -1.20 |
|  | ENSRNOT00000012720 | RGD1307773_predicted | 1.84E-07 | 1.43 |  |  | ENSRNOT00000002394 | Chrd | 7.35E-07 | -1.21 |
|  | ENSRNOT00000059628 | Agrp | 9.79E-07 | 1.43 |  |  | ENSRNOT00000027846 | RGD1308759_predicted | 2.87E-06 | -1.21 |
|  | NM_001004257 | Hspa14 | 4.38E-07 | 1.43 |  |  | AI136617 | AI136617 | 1.36E-07 | -1.21 |
|  | XM_224841 | Odz3_predicted | 1.45E-07 | 1.43 |  |  | NM_031644 | Ptgds2 | 2.88E-07 | -1.21 |
|  | XM_216920 | LOC299907 | 1.08E-07 | 1.43 |  |  | XM_217271 | XM_217271 | 8.67E-07 | -1.21 |
|  | AI059146 | AI059146 | 1.53E-07 | 1.42 |  |  | ENSRNOT00000056191 | RGD1305162_predicted | 1.76E-06 | -1.21 |
|  | TC609617 | TC609617 | 3.51E-07 | 1.42 |  |  | NM_030835 | RAMP4 | 3.49E-05 | -1.21 |
|  | ENSRNOT00000025489 | ENSRNOT00000025489 | 4.97E-06 | 1.42 |  |  | TC613479 | TC613479 | 1.51E-07 | -1.21 |
|  | NM_053896 | Aldh1a2 | 2.70E-07 | 1.42 |  |  | A_44_P229805 | A_44_P229805 | 3.61E-07 | -1.21 |
|  | ENSRNOT00000046191 | ENSRNOT00000046191 | 1.05E-05 | 1.42 |  |  | ENSRNOT00000061891 | Traf2_predicted | 1.45E-06 | -1.21 |
|  | AI231349 | AI231349 | 1.01E-07 | 1.42 |  |  | NM_199097 | Adi1 | 8.98E-06 | -1.21 |
|  | ENSRNOT00000046279 | ENSRNOT00000046279 | 0.000123 | 1.41 |  |  | NM_001024365 | LOC501232 | 3.99E-07 | -1.21 |
|  | XM_001069243 | LOC689030 | 5.50E-07 | 1.41 |  |  | ENSRNOT00000021149 | RGD1560341_predicted | 0.013497 | -1.21 |
|  | XM_576044 | RGD1564008_predicted | 9.52E-08 | 1.41 |  |  | DV726790 | DV726790 | 5.21E-07 | -1.21 |
|  | XM_344307 | XM_344307 | 1.41E-07 | 1.41 |  |  | NM_012960 | Ggh | 1.74E-06 | -1.22 |
|  | NM_198757 | Srr | 8.73E-07 | 1.41 |  |  | NM_139087 | Cgref1 | 2.92E-07 | -1.22 |
|  | NM_021689 | Ereg | 5.30E-07 | 1.40 |  |  | ENSRNOT00000048687 | Srl_predicted | 6.23E-06 | -1.22 |
|  | XM_215841 | XM_215841 | 1.70E-07 | 1.40 |  |  | ENSRNOT00000020021 | RGD1559904_predicted | 1.85E-07 | -1.22 |
|  | BF545883 | BF545883 | 1.41E-05 | 1.40 |  |  | ENSRNOT00000037290 | Bpil2_predicted | 1.83E-07 | -1.22 |
|  | NM_053588 | Rnf138 | 2.68E-07 | 1.40 |  |  | XR_007933 | RGD1560794_predicted | 1.52E-06 | -1.22 |
|  | CD371954 | CD371954 | 9.25E-08 | 1.40 |  |  | NM_001013217 | Trim54 | 3.34E-07 | -1.22 |
|  | A_44_P723917 | A_44_P723917 | 2.44E-05 | 1.40 |  |  | ENSRNOT00000047137 | LOC294844 | 1.03E-06 | -1.22 |
|  | XM_216934 | RGD1561749_predicted | 4.99E-07 | 1.40 |  |  | A_44_P713885 | A_44_P713885 | 1.39E-05 | -1.23 |
|  | TC605762 | TC605762 | 0.000184 | 1.40 |  |  | NM_181631 | Fbxo11 | 0.006674 | -1.23 |
|  | ENSRNOT00000004942 | ENSRNOT00000004942 | 1.24E-06 | 1.40 |  |  | XR_006635 | LOC289673 | 9.12E-06 | -1.23 |
|  | CR753932 | CR753932 | 7.10E-06 | 1.40 |  |  | NM_030866 | Nfix | 3.93E-06 | -1.23 |
|  | NM_001044244 | Tomm34_predicted | 1.33E-05 | 1.40 |  |  | ENSRNOT00000013100 | Poldip2_predicted | 3.59E-06 | -1.23 |
|  | NM_058211 | Slc4a7 | 2.55E-06 | 1.39 |  |  | NM_001008335 | Eif4a2 | 1.36E-05 | -1.23 |
|  | NM_001000387 | Olr416_predicted | 2.29E-06 | 1.39 |  |  | NM_017245 | Eef2 | 1.98E-07 | -1.23 |
|  | ENSRNOT00000007630 | RGD1306484_predicted | 7.88E-08 | 1.39 |  |  | A_44_P630159 | A_44_P630159 | 2.47E-07 | -1.23 |
|  | BI292115 | BI292115 | 1.55E-06 | 1.39 |  |  | NM_023960 | Kcnmb4 | 3.07E-07 | -1.23 |
|  | XR_008061 | RGD1560954_predicted | 2.42E-06 | 1.39 |  |  | NM_139230 | Nexn | 0.000118 | -1.23 |
|  | NM_031511 | Igf2 | 3.66E-07 | 1.39 |  |  | ENSRNOT00000019157 | RGD1564993_predicted | 1.07E-06 | -1.24 |
|  | BF403483 | BF403483 | 4.70E-07 | 1.39 |  |  | BC089922 | Cc2d1b | 1.97E-06 | -1.24 |
|  | XM_001063452 | LOC682861 | 1.05E-07 | 1.38 |  |  | BQ210430 | BQ210430 | 8.12E-05 | -1.24 |
|  | ENSRNOT00000003344 | ENSRNOT00000003344 | 7.60E-07 | 1.38 |  |  | XM_236746 | XM_236746 | 3.63E-06 | -1.24 |
|  | TC602839 | TC602839 | 0.00038 | 1.38 |  |  | NM_001011941 | Cdc37l1 | 0.000448 | -1.24 |
|  | NM_017243 | Prps1 | 5.78E-07 | 1.38 |  |  | NM_022962 | Lphn1 | 5.11E-07 | -1.24 |
|  | NM_022300 | Basp1 | 1.41E-06 | 1.38 |  |  | ENSRNOT00000019329 | Eif4g3_predicted | 8.14E-05 | -1.24 |
|  | AI179012 | AI179012 | 4.63E-06 | 1.38 |  |  | ENSRNOT00000026093 | Ankrd22_predicted | 0.000393 | -1.24 |
|  | A_44_P421738 | A_44_P421738 | 1.49E-05 | 1.38 |  |  | ENSRNOT00000013249 | Bckdhb | 1.19E-06 | -1.24 |
|  | NM_017062 | Ril | 1.58E-06 | 1.38 |  |  | XM_225204 | RGD1307284_predicted | 1.38E-07 | -1.24 |
|  | NM_022541 | Timm8b | 2.54E-07 | 1.37 |  |  | NM_001013080 | Clic3 | 2.02E-07 | -1.24 |
|  | TC612916 | TC612916 | 1.03E-07 | 1.37 |  |  | NM_013003 | Pemt | 1.10E-05 | -1.24 |
|  | NM_031107 | Rps6ka1 | 1.50E-07 | 1.37 |  |  | XM_221686 | Chodl_predicted | 1.89E-06 | -1.24 |
|  | ENSRNOT00000016285 | Kdelc1 | 2.81E-07 | 1.37 |  |  | XM_214085 | XM_214085 | 1.08E-05 | -1.24 |
|  | ENSRNOT00000017889 | RGD1563166_predicted | 1.37E-07 | 1.37 |  |  | NM_001014035 | LOC309957 | 1.88E-06 | -1.24 |
|  | ENSRNOT00000002540 | ENSRNOT00000002540 | 1.30E-07 | 1.37 |  |  | AB012231 | Nfib | 5.26E-07 | -1.24 |
|  | XM_214276 | XM_214276 | 1.03E-07 | 1.37 |  |  | NM_013104 | Igfbp6 | 2.30E-07 | -1.24 |
|  | BF563927 | BF563927 | 2.98E-05 | 1.36 |  |  | NM_012600 | Me1 | 1.04E-07 | -1.24 |
|  | XM_215701 | Papss1_predicted | 5.35E-07 | 1.36 |  |  | NM_019349 | Slk | 7.33E-06 | -1.24 |
|  | XM_233740 | RGD1561264_predicted | 2.17E-07 | 1.36 |  |  | A_44_P389297 | A_44_P389297 | 7.94E-07 | -1.24 |
|  | NM_001013149 | Mesdc1 | 4.56E-07 | 1.36 |  |  | ENSRNOT00000006034 | Brd1_predicted | 1.83E-06 | -1.24 |
|  | AA858639 | AA858639 | 1.46E-06 | 1.36 |  |  | NM_053585 | Madd | 1.18E-07 | -1.25 |
|  | BC099168 | LOC362703 | 2.98E-07 | 1.36 |  |  | AI146186 | AI146186 | 4.45E-07 | -1.25 |
|  | NM_019359 | Cnn3 | 2.49E-07 | 1.36 |  |  | ENSRNOT00000006943 | RGD1564315_predicted | 7.71E-05 | -1.25 |
|  | NM_001012074 | Herc4 | 0.001546 | 1.35 |  |  | NM_024399 | Aspa | 5.86E-05 | -1.25 |
|  | ENSRNOT00000019107 | Armet_predicted | 6.88E-07 | 1.35 |  |  | AW918097 | AW918097 | 1.83E-07 | -1.25 |
|  | ENSRNOT00000002851 | RGD1304621_predicted | 4.92E-07 | 1.35 |  |  | ENSRNOT00000038429 | ENSRNOT00000038429 | 8.84E-07 | -1.25 |
|  | ENSRNOT00000018127 | ENSRNOT00000018127 | 8.28E-08 | 1.35 |  |  | NM_181480 | Nadsyn1 | 4.67E-07 | -1.25 |
|  | XM_235156 | Ptprb_predicted | 7.96E-07 | 1.35 |  |  | NM_001014136 | Ngly1 | 0.000402 | -1.25 |
|  | NM_021583 | Ptges | 1.32E-07 | 1.35 |  |  | TC602194 | TC602194 | 4.57E-05 | -1.25 |
|  | TC632814 | TC632814 | 4.53E-05 | 1.35 |  |  | BC090008 | BC090008 | 2.53E-07 | -1.25 |
|  | ENSRNOT00000018116 | Col9a1 | 7.74E-07 | 1.35 |  |  | AW918767 | AW918767 | 1.59E-07 | -1.26 |
|  | ENSRNOT00000058561 | ENSRNOT00000058561 | 2.00E-07 | 1.35 |  |  | BF405054 | BF405054 | 8.67E-07 | -1.26 |
|  | BQ782195 | BQ782195 | 5.65E-07 | 1.35 |  |  | NM_001000727 | Olr1551_predicted | 2.68E-07 | -1.26 |
|  | NM_019296 | Cdc2a | 3.41E-06 | 1.34 |  |  | ENSRNOT00000027067 | RGD1305020_predicted | 1.24E-06 | -1.26 |
|  | NM_001025705 | Azi2 | 8.19E-07 | 1.34 |  |  | NM_001012179 | Fxr1h | 2.67E-05 | -1.26 |
|  | NM_031588 | Nrg1 | 3.54E-07 | 1.34 |  |  | A_44_P468756 | A_44_P468756 | 6.70E-07 | -1.26 |
|  | XM_001072919 | LOC690012 | 2.53E-06 | 1.34 |  |  | CB548450 | CB548450 | 1.39E-06 | -1.26 |
|  | NM_021838 | Nos3 | 2.24E-06 | 1.34 |  |  | AI639532 | AI639532 | 0.000285 | -1.26 |
|  | TC582175 | TC582175 | 9.82E-08 | 1.34 |  |  | ENSRNOT00000037176 | Ndufa9 | 2.62E-06 | -1.26 |
|  | DQ901406 | LOC680080 | 1.28E-07 | 1.34 |  |  | XM_217354 | XM_217354 | 2.21E-06 | -1.26 |
|  | NM_053667 | Lepre1 | 2.48E-07 | 1.34 |  |  | ENSRNOT00000027244 | LOC682386 | 6.59E-06 | -1.26 |
|  | NM_031633 | Foxm1 | 3.44E-07 | 1.34 |  |  | XM_221736 | XM_221736 | 0.01298 | -1.26 |
|  | A_44_P133672 | A_44_P133672 | 9.09E-07 | 1.34 |  |  | NM_001007664 | Abhd14b | 2.95E-07 | -1.26 |
|  | ENSRNOT00000013738 | Tmem9_predicted | 3.77E-07 | 1.34 |  |  | NM_001014199 | Atp6v1c2 | 3.48E-06 | -1.27 |
|  | NM_013185 | Hck | 2.17E-07 | 1.34 |  |  | NM_053581 | Idh3B | 0.000149 | -1.27 |
|  | ENSRNOT00000027404 | ENSRNOT00000027404 | 9.10E-07 | 1.34 |  |  | NM_022618 | Akap6 | 1.65E-06 | -1.27 |
|  | AI030078 | AI030078 | 9.75E-08 | 1.34 |  |  | AW525193 | AW525193 | 3.57E-06 | -1.27 |
|  | XM_001065777 | LOC685924 | 3.75E-07 | 1.34 |  |  | NM_001008804 | Krt10 | 2.60E-06 | -1.27 |
|  | XR_005908 | LOC365047 | 5.61E-07 | 1.33 |  |  | NM_012774 | Gpc3 | 2.83E-05 | -1.27 |
|  | AA860061 | AA860061 | 1.67E-07 | 1.33 |  |  | NM_133621 | Hod | 0.000141 | -1.27 |
|  | AI227710 | AI227710 | 3.16E-07 | 1.33 |  |  | A_44_P775809 | A_44_P775809 | 6.78E-07 | -1.27 |
|  | ENSRNOT00000008136 | RGD1309019_predicted | 9.74E-08 | 1.33 |  |  | ENSRNOT00000032663 | Cldn17_predicted | 1.47E-07 | -1.27 |
|  | AY539878 | RGD1311678 | 4.22E-07 | 1.33 |  |  | ENSRNOT00000004064 | ENSRNOT00000004064 | 4.11E-06 | -1.27 |
|  | A_44_P204173 | A_44_P204173 | 2.97E-07 | 1.33 |  |  | AW918768 | AW918768 | 9.95E-06 | -1.27 |
|  | TC617265 | TC617265 | 9.70E-08 | 1.33 |  |  | AA900593 | AA900593 | 0.002033 | -1.27 |
|  | CF110190 | CF110190 | 1.25E-06 | 1.33 |  |  | DV725602 | DV725602 | 1.21E-07 | -1.28 |
|  | NM_198789 | Bzw1 | 1.23E-06 | 1.33 |  |  | NM_138877 | Cyb5r3 | 4.87E-07 | -1.28 |
|  | ENSRNOT00000008115 | RGD1306402 | 0.000144 | 1.33 |  |  | NM_031840 | Fdps | 6.90E-06 | -1.28 |
|  | ENSRNOT00000060594 | Ccl27_predicted | 1.71E-06 | 1.33 |  |  | ENSRNOT00000049582 | ENSRNOT00000049582 | 2.21E-07 | -1.28 |
|  | DV718743 | DV718743 | 1.40E-07 | 1.32 |  |  | XM_001073426 | LOC690139 | 1.44E-05 | -1.28 |
|  | A_44_P255965 | A_44_P255965 | 6.08E-07 | 1.32 |  |  | ENSRNOT00000025638 | Egfl9_predicted | 1.16E-07 | -1.28 |
|  | AA900505 | AA900505 | 9.99E-07 | 1.32 |  |  | ENSRNOT00000051653 | Aggf1 | 9.31E-08 | -1.28 |
|  | NM_012517 | Cacna1c | 2.59E-07 | 1.32 |  |  | AA900409 | AA900409 | 0.000552 | -1.28 |
|  | XM_001064901 | LOC688540 | 4.49E-07 | 1.32 |  |  | NM_001011941 | Cdc37l1 | 0.001628 | -1.28 |
|  | CN540809 | CN540809 | 0.003425 | 1.32 |  |  | NM_022282 | Dlgh2 | 1.38E-07 | -1.28 |
|  | NM_001024257 | RGD1309383 | 1.35E-06 | 1.32 |  |  | NM_001013082 | Pon2 | 0.007492 | -1.28 |
|  | ENSRNOT00000013790 | ENSRNOT00000013790 | 7.30E-06 | 1.32 |  |  | NM_001013112 | Hibch | 0.001544 | -1.29 |
|  | BM384260 | BM384260 | 0.000167 | 1.32 |  |  | NM_022262 | Tsnax | 2.58E-05 | -1.29 |
|  | A_44_P629540 | A_44_P629540 | 6.68E-05 | 1.32 |  |  | NM_181550 | Sqstm1 | 1.28E-07 | -1.29 |
|  | BF287427 | BF287427 | 5.67E-05 | 1.32 |  |  | XM_233945 | LOC313934 | 8.45E-06 | -1.29 |
|  | AW143690 | AW143690 | 2.55E-07 | 1.31 |  |  | NM_001007622 | Pdlim2 | 8.09E-08 | -1.29 |
|  | NM_022600 | Adcy5 | 2.45E-07 | 1.31 |  |  | AI136185 | AI136185 | 0.000294 | -1.29 |
|  | ENSRNOT00000002779 | Ifngr2_predicted | 4.67E-07 | 1.31 |  |  | TC598143 | TC598143 | 0.00634 | -1.30 |
|  | ENSRNOT00000005835 | Pole2_predicted | 1.44E-05 | 1.30 |  |  | ENSRNOT00000043415 | ENSRNOT00000043415 | 2.55E-06 | -1.30 |
|  | AW532988 | AW532988 | 5.40E-05 | 1.30 |  |  | ENSRNOT00000010630 | ENSRNOT00000010630 | 9.45E-08 | -1.30 |
|  | XM_345920 | RGD1563738_predicted | 4.49E-07 | 1.30 |  |  | ENSRNOT00000060718 | ENSRNOT00000060718 | 0.000509 | -1.30 |
|  | NM_001024303 | LOC499677 | 6.32E-07 | 1.30 |  |  | ENSRNOT00000005653 | Spag7_predicted | 0.000147 | -1.30 |
|  | NM_013219 | Cadps | 1.97E-06 | 1.30 |  |  | NM_001044231 | RGD1562305_predicted | 3.42E-06 | -1.30 |
|  | BE119515 | BE119515 | 4.76E-07 | 1.30 |  |  | ENSRNOT00000015152 | Hnrpa2b1_predicted | 3.51E-05 | -1.30 |
|  | ENSRNOT00000016652 | Mybpc3_predicted | 1.60E-05 | 1.30 |  |  | XM_218313 | Gpr126_predicted | 1.14E-06 | -1.30 |
|  | ENSRNOT00000059467 | RGD1565641_predicted | 2.94E-06 | 1.30 |  |  | NM_017276 | Gdi2 | 0.014065 | -1.31 |
|  | NM_020091 | LOC56825 | 1.78E-07 | 1.30 |  |  | ENSRNOT00000025501 | RGD1564854_predicted | 6.27E-07 | -1.31 |
|  | TC610139 | TC610139 | 0.001885 | 1.29 |  |  | NM_001044275 | LOC679532 | 7.87E-06 | -1.31 |
|  | AI045896 | AI045896 | 6.58E-06 | 1.29 |  |  | ENSRNOT00000031504 | Calm4_predicted | 1.37E-07 | -1.31 |
|  | NM_001000313 | Olr513_predicted | 2.06E-07 | 1.29 |  |  | ENSRNOT00000014583 | Phr1_predicted | 9.57E-05 | -1.31 |
|  | NM_053685 | Hcn3 | 1.04E-06 | 1.29 |  |  | NM_001001272 | Olr130_predicted | 8.49E-08 | -1.31 |
|  | ENSRNOT00000021723 | RGD1560953_predicted | 2.82E-06 | 1.29 |  |  | AB012231 | Nfib | 4.61E-06 | -1.31 |
|  | ENSRNOT00000020999 | RGD1565844_predicted | 4.68E-07 | 1.29 |  |  | ENSRNOT00000016804 | Zfp91 | 4.48E-05 | -1.31 |
|  | ENSRNOT00000019862 | Iars_predicted | 3.20E-07 | 1.29 |  |  | NM_031347 | Ppargc1a | 0.000537 | -1.31 |
|  | NM_001008554 | RGD1306446 | 8.84E-06 | 1.29 |  |  | NM_133586 | Ces2 | 2.57E-07 | -1.31 |
|  | NM_182949 | Prss27 | 1.91E-07 | 1.29 |  |  | NM_001000585 | Olr859_predicted | 3.64E-07 | -1.31 |
|  | BE113698 | BE113698 | 1.00E-06 | 1.28 |  |  | NM_012595 | Ldhb | 1.54E-06 | -1.31 |
|  | NM_012551 | Egr1 | 4.19E-07 | 1.28 |  |  | XR_007169 | LOC293458 | 0.000109 | -1.32 |
|  | XM_576510 | RGD1563347_predicted | 4.16E-07 | 1.28 |  |  | ENSRNOT00000042097 | Rnf31_predicted | 3.94E-06 | -1.32 |
|  | ENSRNOT00000004702 | Hcfc1r1 | 2.74E-07 | 1.28 |  |  | ENSRNOT00000055831 | ENSRNOT00000055831 | 8.62E-08 | -1.32 |
|  | AI012613 | AI012613 | 3.38E-06 | 1.28 |  |  | ENSRNOT00000024128 | Sfrp1 | 1.29E-05 | -1.32 |
|  | X05566 | Mrlcb | 1.75E-07 | 1.28 |  |  | AY724520 | LOC690085 | 0.000248 | -1.32 |
|  | NM_012678 | Tpm4 | 6.22E-07 | 1.27 |  |  | NM_031338 | Camkk2 | 1.54E-07 | -1.32 |
|  | NM_001025673 | LOC306464 | 8.17E-06 | 1.27 |  |  | ENSRNOT00000023364 | ENSRNOT00000023364 | 2.54E-07 | -1.32 |
|  | XM_001066790 | LOC683626 | 1.81E-07 | 1.27 |  |  | XR_005611 | LOC315534 | 1.60E-07 | -1.32 |
|  | XM_001057993 | LOC680611 | 4.53E-07 | 1.27 |  |  | ENSRNOT00000019265 | Arpc2_predicted | 1.86E-06 | -1.32 |
|  | XM_221962 | Ttyh3_predicted | 5.60E-07 | 1.27 |  |  | ENSRNOT00000008459 | RGD1561653_predicted | 6.64E-06 | -1.33 |
|  | ENSRNOT00000004942 | ENSRNOT00000004942 | 1.05E-06 | 1.27 |  |  | NM_022865 | Gphn | 1.59E-06 | -1.33 |
|  | AI639455 | AI639455 | 2.74E-05 | 1.26 |  |  | BC085903 | BC085903 | 1.15E-05 | -1.33 |
|  | ENSRNOT00000036556 | LOC501052 | 3.11E-06 | 1.26 |  |  | NM_001009409 | Nanp | 0.000306 | -1.33 |
|  | ENSRNOT00000036082 | RGD1310645_predicted | 1.23E-07 | 1.26 |  |  | XR_008801 | Elmod1_predicted | 0.000404 | -1.34 |
|  | ENSRNOT00000038162 | ENSRNOT00000038162 | 9.35E-07 | 1.25 |  |  | NM_031777 | Usf1 | 1.62E-05 | -1.34 |
|  | NM_033234 | Hbb | 1.57E-05 | 1.25 |  |  | ENSRNOT00000041209 | ENSRNOT00000041209 | 2.23E-07 | -1.34 |
|  | NM_001013897 | LOC293103 | 5.21E-07 | 1.25 |  |  | ENSRNOT00000004783 | Smyd2 | 2.24E-07 | -1.34 |
|  | AW915986 | AW915986 | 1.03E-06 | 1.25 |  |  | BF555763 | BF555763 | 2.20E-06 | -1.34 |
|  | ENSRNOT00000018470 | Ric8a | 2.24E-07 | 1.25 |  |  | TC629976 | TC629976 | 1.05E-07 | -1.34 |
|  | NM_199387 | Mterfd1 | 3.54E-07 | 1.25 |  |  | ENSRNOT00000007548 | RGD1308796_predicted | 5.13E-07 | -1.34 |
|  | ENSRNOT00000043702 | ENSRNOT00000043702 | 9.86E-05 | 1.25 |  |  | NM_138905 | Ppap2b | 2.02E-07 | -1.34 |
|  | NM_019259 | C1qbp | 1.61E-06 | 1.25 |  |  | XM_226181 | XM_226181 | 6.06E-06 | -1.34 |
|  | DV721627 | DV721627 | 1.64E-06 | 1.25 |  |  | NM_080905 | Siah1a | 0.000143 | -1.35 |
|  | NM_019190 | Mcp | 2.56E-07 | 1.24 |  |  | ENSRNOT00000014800 | RGD1311340_predicted | 4.03E-07 | -1.35 |
|  | XM_001060373 | LOC682199 | 1.10E-06 | 1.24 |  |  | NM_012923 | Ccng1 | 3.30E-05 | -1.35 |
|  | XM_001059639 | LOC681383 | 1.10E-06 | 1.24 |  |  | NM_021758 | Lin7b | 4.58E-07 | -1.35 |
|  | NM_053927 | Epb4.1l3 | 2.87E-07 | 1.24 |  |  | NM_031152 | Rab11a | 9.09E-05 | -1.35 |
|  | NM_031142 | Doc2b | 4.28E-07 | 1.24 |  |  | AW143174 | AW143174 | 0.002243 | -1.35 |
|  | BF406830 | BF406830 | 5.04E-07 | 1.24 |  |  | DV721486 | DV721486 | 0.000124 | -1.35 |
|  | BE098727 | BE098727 | 0.00011 | 1.24 |  |  | AA955043 | AA955043 | 1.96E-07 | -1.35 |
|  | BF407981 | BF407981 | 2.36E-07 | 1.24 |  |  | NM_053713 | Klf4 | 3.22E-06 | -1.35 |
|  | TC598283 | TC598283 | 2.03E-07 | 1.24 |  |  | NM_031576 | Por | 1.30E-07 | -1.35 |
|  | NM_017100 | Plk1 | 3.54E-07 | 1.24 |  |  | XR_007553 | RGD1562187_predicted | 1.73E-05 | -1.36 |
|  | NM_013049 | Tnfrsf4 | 3.35E-07 | 1.24 |  |  | NM_130412 | Sdf4 | 1.70E-05 | -1.36 |
|  | NM_017019 | Il1a | 7.19E-05 | 1.24 |  |  | XM_345051 | XM_345051 | 3.64E-05 | -1.36 |
|  | NM_053862 | Lgals8 | 3.26E-06 | 1.24 |  |  | NM_001000849 | Olr809_predicted | 1.37E-07 | -1.36 |
|  | AI029806 | AI029806 | 2.97E-07 | 1.24 |  |  | NM_199085 | Serpinb6a | 6.93E-05 | -1.36 |
|  | NM_001014258 | RGD1311037 | 2.18E-07 | 1.23 |  |  | NM_144562 | Fmo4 | 0.000668 | -1.36 |
|  | NM_019630 | Gip | 1.85E-07 | 1.23 |  |  | ENSRNOT00000019331 | ENSRNOT00000019331 | 4.19E-06 | -1.36 |
|  | BG665051 | BG665051 | 8.31E-06 | 1.23 |  |  | NM_001011959 | Ppgb | 2.07E-07 | -1.36 |
|  | BM390141 | BM390141 | 2.59E-07 | 1.23 |  |  | DV723169 | DV723169 | 1.55E-06 | -1.36 |
|  | TC617559 | TC617559 | 0.001678 | 1.23 |  |  | XM_225097 | XM_225097 | 6.70E-06 | -1.36 |
|  | ENSRNOT00000002970 | Pvrl3_predicted | 7.18E-07 | 1.23 |  |  | NM_001024964 | Exoc3 | 3.86E-05 | -1.37 |
|  | NM_001008330 | Rpo1-1 | 3.55E-07 | 1.23 |  |  | XM_224540 | XM_224540 | 3.61E-06 | -1.37 |
|  | NM_053963 | Mmp12 | 7.14E-07 | 1.23 |  |  | NM_145775 | Nr1d1 | 4.38E-07 | -1.37 |
|  | NM_001008761 | Krt1-12 | 6.02E-06 | 1.23 |  |  | XM_213403 | RGD1309400_predicted | 1.78E-07 | -1.38 |
|  | AA850372 | AA850372 | 0.000245 | 1.23 |  |  | NM_053772 | Pkia | 8.94E-05 | -1.38 |
|  | XR_009176 | LOC687293 | 2.32E-06 | 1.23 |  |  | ENSRNOT00000029177 | ENSRNOT00000029177 | 0.002401 | -1.38 |
|  | ENSRNOT00000046319 | ENSRNOT00000046319 | 4.09E-06 | 1.23 |  |  | ENSRNOT00000006415 | Pof1b_predicted | 7.19E-07 | -1.38 |
|  | ENSRNOT00000004939 | RGD1559740_predicted | 3.37E-07 | 1.22 |  |  | XM_215119 | Rassf7_predicted | 8.06E-08 | -1.39 |
|  | AY310157 | RGD1311563 | 1.63E-06 | 1.22 |  |  | A_44_P667748 | A_44_P667748 | 2.07E-05 | -1.39 |
|  | NM_001009627 | Yars2 | 1.83E-07 | 1.22 |  |  | ENSRNOT00000008159 | RGD1562562_predicted | 4.56E-05 | -1.39 |
|  | BF522189 | BF522189 | 3.50E-06 | 1.22 |  |  | XR_007139 | LOC364713 | 7.18E-06 | -1.39 |
|  | ENSRNOT00000033720 | ENSRNOT00000033720 | 0.000216 | 1.22 |  |  | ENSRNOT00000018435 | Clca2_predicted | 6.30E-07 | -1.39 |
|  | AA998448 | AA998448 | 3.56E-07 | 1.22 |  |  | NM_153297 | Cops2 | 0.005466 | -1.39 |
|  | ENSRNOT00000051365 | ENSRNOT00000051365 | 1.92E-06 | 1.22 |  |  | AI113137 | AI113137 | 7.26E-08 | -1.39 |
|  | NM_017180 | Phlda1 | 4.95E-07 | 1.22 |  |  | TC616443 | TC616443 | 5.43E-05 | -1.40 |
|  | NM_001014274 | Armcx2 | 1.02E-06 | 1.22 |  |  | NM_001007759 | 03-Mar | 2.63E-07 | -1.40 |
|  | ENSRNOT00000032092 | Slc5a10_predicted | 2.03E-07 | 1.22 |  |  | AW915015 | AW915015 | 2.34E-07 | -1.40 |
|  | TC611540 | TC611540 | 0.000498 | 1.22 |  |  | NM_053416 | Strbp | 0.000109 | -1.40 |
|  | AA817887 | AA817887 | 3.48E-07 | 1.22 |  |  | ENSRNOT00000025641 | Ankrd13d_predicted | 3.12E-07 | -1.40 |
|  | NM_022860 | B4galnt1 | 1.83E-06 | 1.22 |  |  | NM_022543 | Ssg1 | 0.000139 | -1.41 |
|  | NM_139096 | Lgals3bp | 8.87E-07 | 1.21 |  |  | BI302132 | BI302132 | 9.21E-08 | -1.41 |
|  | ENSRNOT00000038123 | Zfp692_predicted | 3.90E-07 | 1.21 |  |  | ENSRNOT00000034884 | ENSRNOT00000034884 | 4.41E-06 | -1.41 |
|  | ENSRNOT00000056307 | Plxna3 | 7.79E-07 | 1.21 |  |  | NM_013159 | Ide | 8.70E-08 | -1.41 |
|  | NM_001034911 | RGD1306614 | 9.15E-07 | 1.21 |  |  | NM_139336 | Uxs1 | 0.000287 | -1.41 |
|  | ENSRNOT00000014519 | RGD1311530_predicted | 8.96E-07 | 1.21 |  |  | NM_001025695 | Cops5 | 0.001541 | -1.41 |
|  | NM_201422 | Pcdhac2 | 9.86E-07 | 1.21 |  |  | A_44_P229451 | A_44_P229451 | 7.80E-07 | -1.41 |
|  | XM_340798 | P4ha2_predicted | 8.66E-07 | 1.21 |  |  | XR_008245 | RGD1565176_predicted | 1.08E-07 | -1.42 |
|  | ENSRNOT00000027180 | Crlf1_predicted | 2.80E-07 | 1.21 |  |  | NM_173105 | Aqp11 | 5.16E-08 | -1.42 |
|  | ENSRNOT00000006999 | RGD1561582_predicted | 5.26E-06 | 1.21 |  |  | ENSRNOT00000029583 | Myom1 | 8.09E-08 | -1.42 |
|  | NM_031802 | Gabbr2 | 7.20E-07 | 1.21 |  |  | NM_032416 | Aldh2 | 2.01E-07 | -1.42 |
|  | TC614795 | TC614795 | 4.88E-07 | 1.21 |  |  | NM_017172 | Zfp36l1 | 0.005388 | -1.42 |
|  | ENSRNOT00000020457 | ENSRNOT00000020457 | 4.44E-07 | 1.21 |  |  | NM_001007742 | Ubadc1 | 1.44E-06 | -1.42 |
|  | BF391602 | RGD1559578_predicted | 3.38E-05 | 1.20 |  |  | BF289404 | BF289404 | 2.26E-07 | -1.42 |
|  | AI169327 | AI169327 | 2.35E-06 | 1.20 |  |  | ENSRNOT00000056437 | Tuft1_predicted | 1.29E-07 | -1.43 |
|  | ENSRNOT00000036748 | Wdr41_predicted | 4.01E-05 | 1.20 |  |  | AF036548 | Rgc32 | 7.80E-08 | -1.43 |
|  | NM_024484 | Alas1 | 4.14E-07 | 1.20 |  |  | XM_575393 | RGD1559697_predicted | 7.92E-06 | -1.43 |
|  | NM_001004215 | Ppic | 2.90E-07 | 1.20 |  |  | NM_001042354 | Camk2b | 6.40E-08 | -1.43 |
|  | BI303604 | BI303604 | 3.59E-05 | 1.20 |  |  | ENSRNOT00000000365 | Hace1_predicted | 0.00439 | -1.43 |
|  | NM_181086 | Tnfrsf12a | 9.02E-07 | 1.20 |  |  | NM_022865 | Gphn | 5.53E-08 | -1.43 |
|  | XR_007429 | RGD1563029_predicted | 7.95E-07 | 1.20 |  |  | ENSRNOT00000019802 | RGD1306148_predicted | 7.37E-06 | -1.43 |
|  | NM_053997 | Kcnc3 | 9.15E-07 | 1.20 |  |  | NM_001031639 | Psmd2 | 3.30E-05 | -1.44 |
|  | NM_012915 | Atpif1 | 1.37E-06 | 1.20 |  |  | NM_138863 | Ltb4dh | 4.14E-08 | -1.44 |
|  | ENSRNOT00000019374 | RGD1566242_predicted | 3.96E-05 | 1.20 |  |  | AY325254 | Da2-19 | 0.000968 | -1.44 |
|  | ENSRNOT00000019273 | Zic5_predicted | 8.61E-06 | 1.19 |  |  | AI410543 | AI410543 | 4.94E-08 | -1.44 |
|  | NM_031631 | Vapa | 5.65E-05 | 1.19 |  |  | TC620427 | TC620427 | 2.57E-06 | -1.44 |
|  | NM_001014201 | Cd320 | 1.88E-07 | 1.19 |  |  | NM_031816 | Rbbp7 | 0.002582 | -1.44 |
|  | NM_031056 | Mmp14 | 1.44E-05 | 1.19 |  |  | XM_573664 | RGD1566234_predicted | 1.82E-06 | -1.45 |
|  | ENSRNOT00000037724 | ENSRNOT00000037724 | 0.000221 | 1.19 |  |  | XR_006910 | LOC288481 | 3.34E-07 | -1.45 |
|  | NM_017006 | G6pdx | 2.72E-07 | 1.19 |  |  | NM_001014208 | Yipf2 | 1.57E-07 | -1.45 |
|  | ENSRNOT00000040993 | ND2 | 5.75E-07 | 1.19 |  |  | ENSRNOT00000036996 | RGD1560395_predicted | 5.93E-06 | -1.45 |
|  | NM_012759 | Vav1 | 1.18E-06 | 1.19 |  |  | NM_017141 | Polb | 4.11E-06 | -1.45 |
|  | BQ208148 | BQ208148 | 8.21E-05 | 1.19 |  |  | NM_012815 | Gclc | 0.000314 | -1.46 |
|  | ENSRNOT00000000471 | Asf1a_predicted | 3.68E-06 | 1.19 |  |  | ENSRNOT00000028196 | ENSRNOT00000028196 | 4.93E-08 | -1.46 |
|  | NM_153294 | LOC259224 | 0.000436 | 1.19 |  |  | ENSRNOT00000043998 | RGD1563599_predicted | 3.59E-07 | -1.46 |
|  | ENSRNOT00000016797 | Aspm_predicted | 6.77E-07 | 1.19 |  |  | NM_001037097 | Pomt2 | 4.26E-06 | -1.46 |
|  | ENSRNOT00000003452 | Tnn_predicted | 1.19E-05 | 1.18 |  |  | XM_001055377 | LOC679379 | 5.65E-05 | -1.46 |
|  | ENSRNOT00000010623 | ENSRNOT00000010623 | 5.73E-07 | 1.18 |  |  | NM_053796 | F11r | 6.70E-06 | -1.46 |
|  | NM_001017537 | Tex261 | 1.99E-07 | 1.18 |  |  | NM_001009920 | Yc2 | 1.55E-06 | -1.46 |
|  | XM_222160 | XM_222160 | 3.29E-06 | 1.18 |  |  | XM_001076022 | LOC686853 | 4.23E-06 | -1.46 |
|  | NM_032085 | Col3a1 | 0.000298 | 1.18 |  |  | ENSRNOT00000023245 | Trex2_predicted | 1.08E-07 | -1.47 |
|  | NM_012828 | Cacnb3 | 6.54E-07 | 1.18 |  |  | ENSRNOT00000051718 | Dtna_predicted | 3.19E-07 | -1.47 |
|  | NM_031120 | Ssr3 | 3.40E-07 | 1.18 |  |  | ENSRNOT00000037601 | Nebl_predicted | 5.08E-07 | -1.47 |
|  | NM_053666 | Dll3 | 2.34E-07 | 1.18 |  |  | ENSRNOT00000002150 | Usp25_predicted | 0.001072 | -1.47 |
|  | XM_343031 | RGD1560938_predicted | 2.98E-07 | 1.18 |  |  | NM_001013115 | Guk1 | 6.99E-07 | -1.47 |
|  | NM_001033655 | Dnah1 | 5.14E-06 | 1.18 |  |  | NM_001001506 | Mrgprd | 3.65E-07 | -1.47 |
|  | NM_001014253 | Selt | 3.62E-06 | 1.18 |  |  | NM_031146 | Arpc1a | 6.73E-07 | -1.47 |
|  | NM_130403 | Ppp1r14a | 8.62E-06 | 1.18 |  |  | NM_053326 | Pdlim5 | 5.12E-08 | -1.47 |
|  | ENSRNOT00000018844 | Matn4_predicted | 5.10E-07 | 1.18 |  |  | NM_001012355 | Senp8 | 7.64E-05 | -1.48 |
|  | NM_013197 | Alas2 | 3.08E-07 | 1.18 |  |  | NM_017348 | Slc6a8 | 4.61E-07 | -1.48 |
|  | ENSRNOT00000046363 | ENSRNOT00000046363 | 0.000603 | 1.18 |  |  | BF406314 | BF406314 | 1.71E-06 | -1.48 |
|  | AA997980 | AA997980 | 1.25E-06 | 1.17 |  |  | NM_001012345 | Dgat2 | 4.98E-08 | -1.48 |
|  | ENSRNOT00000049021 | ENSRNOT00000049021 | 4.25E-07 | 1.17 |  |  | NM_012592 | Ivd | 2.13E-07 | -1.48 |
|  | A_44_P822051 | A_44_P822051 | 3.94E-05 | 1.17 |  |  | A_44_P255875 | A_44_P255875 | 2.49E-07 | -1.48 |
|  | NM_001008372 | Papd4 | 6.62E-06 | 1.17 |  |  | XM_218447 | RGD1561121_predicted | 2.96E-07 | -1.48 |
|  | NM_019622 | Espn | 2.02E-06 | 1.17 |  |  | ENSRNOT00000000890 | RGD1309562_predicted | 2.60E-07 | -1.48 |
|  | AA818280 | AA818280 | 4.59E-06 | 1.17 |  |  | AI230598 | AI230598 | 0.000232 | -1.48 |
|  | NM_012656 | Sparc | 4.07E-07 | 1.17 |  |  | NM_001013039 | Art5 | 1.32E-07 | -1.48 |
|  | NM_031628 | Nr4a3 | 5.35E-07 | 1.17 |  |  | NM_012777 | Apod | 1.98E-05 | -1.48 |
|  | NM_001013976 | RGD1305007 | 5.23E-07 | 1.17 |  |  | NM_173125 | Pdlim7 | 6.28E-07 | -1.48 |
|  | ENSRNOT00000037340 | ENSRNOT00000037340 | 2.71E-07 | 1.16 |  |  | NM_017158 | Cyp2c7 | 3.04E-08 | -1.48 |
|  | AA946313 | AA946313 | 1.09E-06 | 1.16 |  |  | XM_344812 | RGD1562948_predicted | 1.68E-06 | -1.49 |
|  | NM_172333 | Cthrc1 | 8.58E-06 | 1.16 |  |  | TC583473 | TC583473 | 7.09E-08 | -1.49 |
|  | BF523428 | BF523428 | 1.19E-06 | 1.16 |  |  | XR_005551 | LOC294903 | 4.76E-06 | -1.49 |
|  | ENSRNOT00000024796 | Racgap1_predicted | 4.17E-07 | 1.16 |  |  | NM_001017466 | MGC108776 | 3.72E-08 | -1.49 |
|  | ENSRNOT00000005961 | Adss_predicted | 4.79E-07 | 1.16 |  |  | XM_340876 | RGD1560397_predicted | 2.97E-06 | -1.49 |
|  | NM_053538 | Laptm5 | 1.21E-06 | 1.16 |  |  | AI555010 | AI555010 | 3.41E-08 | -1.49 |
|  | NM_012797 | Id1 | 3.03E-07 | 1.16 |  |  | NM_031085 | Prkch | 5.82E-06 | -1.49 |
|  | NM_181635 | Kif15 | 2.26E-06 | 1.16 |  |  | NM_017306 | Dci | 3.28E-06 | -1.49 |
|  | ENSRNOT00000007328 | Tspan9_predicted | 2.34E-07 | 1.16 |  |  | NM_053999 | Ppp2r2a | 0.002693 | -1.49 |
|  | NM_031622 | Mapk6 | 1.00E-06 | 1.16 |  |  | NM_022936 | Ephx2 | 5.82E-07 | -1.50 |
|  | CB743542 | CB743542 | 8.01E-06 | 1.16 |  |  | NM_012542 | Cyp2a3a | 4.92E-08 | -1.50 |
|  | NM_022391 | Pttg1 | 1.15E-06 | 1.16 |  |  | XM_215641 | Tmod4_predicted | 1.05E-07 | -1.50 |
|  | BF409331 | BF409331 | 1.40E-06 | 1.16 |  |  | TC587724 | TC587724 | 9.22E-07 | -1.50 |
|  | ENSRNOT00000039759 | Kcnmb3_predicted | 6.55E-07 | 1.16 |  |  | TC619137 | TC619137 | 5.27E-08 | -1.50 |
|  | AI411329 | AI411329 | 5.07E-07 | 1.16 |  |  | XR_005872 | LOC308706 | 1.19E-07 | -1.50 |
|  | NM_019142 | Prkaa1 | 2.62E-05 | 1.15 |  |  | TC606021 | TC606021 | 6.89E-08 | -1.50 |
|  | NM_001044770 | Cyp4a11 | 5.46E-06 | 1.15 |  |  | NM_130433 | Acaa2 | 4.57E-06 | -1.50 |
|  | NM_022500 | Ftl1 | 2.93E-06 | 1.15 |  |  | ENSRNOT00000048557 | RGD1565323_predicted | 1.74E-07 | -1.50 |
|  | XR_009565 | RGD1562181_predicted | 2.75E-07 | 1.15 |  |  | XR_005954 | LOC297785 | 7.77E-07 | -1.51 |
|  | NM_012488 | A2m | 1.53E-06 | 1.15 |  |  | ENSRNOT00000044887 | Cilp_predicted | 6.23E-08 | -1.51 |
|  | NM_001024273 | Cmah | 7.19E-07 | 1.15 |  |  | ENSRNOT00000016163 | Fxr2h_predicted | 2.30E-07 | -1.51 |
|  | NM_031818 | Clic4 | 1.82E-06 | 1.15 |  |  | ENSRNOT00000054733 | Cyp2c65_predicted | 6.12E-05 | -1.51 |
|  | AA925099 | AA925099 | 4.30E-05 | 1.15 |  |  | NM_053407 | Asah1 | 0.00461 | -1.51 |
|  | NM_130428 | Sdha | 1.42E-06 | 1.15 |  |  | NM_001017458 | Tcp11l2 | 0.000735 | -1.51 |
|  | ENSRNOT00000033705 | ENSRNOT00000033705 | 6.31E-07 | 1.15 |  |  | ENSRNOT00000061139 | RGD1307395 | 4.07E-06 | -1.51 |
|  | ENSRNOT00000050327 | ENSRNOT00000050327 | 1.86E-06 | 1.15 |  |  | ENSRNOT00000014604 | Braf | 8.40E-06 | -1.51 |
|  | ENSRNOT00000041423 | ENSRNOT00000041423 | 2.42E-07 | 1.15 |  |  | NM_021699 | Mark2 | 4.33E-07 | -1.51 |
|  | AF217591 | AF217591 | 3.95E-06 | 1.15 |  |  | ENSRNOT00000010097 | RGD1310433_predicted | 3.40E-07 | -1.52 |
|  | NM_030847 | Emp3 | 7.68E-07 | 1.15 |  |  | NM_001007750 | Chpt1 | 5.22E-06 | -1.52 |
|  | ENSRNOT00000042564 | ENSRNOT00000042564 | 0.000915 | 1.15 |  |  | XR_006710 | LOC500330 | 1.08E-07 | -1.52 |
|  | XM_001055992 | LOC680172 | 5.44E-07 | 1.15 |  |  | XM_340847 | XM_340847 | 1.05E-07 | -1.52 |
|  | ENSRNOT00000049759 | Me2_predicted | 1.47E-05 | 1.14 |  |  | XR_008902 | RGD1561911_predicted | 8.13E-08 | -1.52 |
|  | NM_012894 | Adarb1 | 0.000493 | 1.14 |  |  | XR_006468 | LOC299213 | 3.15E-06 | -1.52 |
|  | XM_221343 | RGD1305614_predicted | 3.65E-07 | 1.14 |  |  | ENSRNOT00000056257 | ENSRNOT00000056257 | 2.92E-07 | -1.52 |
|  | AA945910 | AA945910 | 0.001273 | 1.14 |  |  | NM_001007622 | Pdlim2 | 4.21E-08 | -1.52 |
|  | NM_022501 | Crip2 | 6.73E-07 | 1.14 |  |  | ENSRNOT00000021357 | Centb1_predicted | 1.85E-07 | -1.52 |
|  | NM_001025738 | Fusip1 | 4.80E-07 | 1.14 |  |  | A_44_P274772 | A_44_P274772 | 0.00025 | -1.53 |
|  | TC626696 | TC626696 | 0.000382 | 1.14 |  |  | NM_001013164 | Ccbl1 | 2.60E-07 | -1.53 |
|  | XM_216386 | Ctnnal1_predicted | 1.65E-05 | 1.14 |  |  | NM_001011997 | Tmod3 | 8.63E-06 | -1.53 |
|  | ENSRNOT00000035635 | Ncf2_predicted | 4.69E-07 | 1.14 |  |  | A_44_P913975 | A_44_P913975 | 3.91E-07 | -1.53 |
|  | A_44_P220372 | A_44_P220372 | 3.01E-07 | 1.14 |  |  | ENSRNOT00000061504 | Kctd1 | 2.00E-06 | -1.53 |
|  | ENSRNOT00000049464 | ENSRNOT00000049464 | 0.000411 | 1.14 |  |  | NM_012919 | Cacna2d1 | 4.50E-07 | -1.53 |
|  | NM_022797 | Grin2d | 0.000262 | 1.13 |  |  | XM_220863 | XM_220863 | 1.16E-07 | -1.53 |
|  | NM_145094 | Rab31 | 1.92E-05 | 1.13 |  |  | NM_001011927 | Atp6v0d1 | 4.36E-06 | -1.53 |
|  | NM_031677 | Fhl2 | 5.88E-07 | 1.13 |  |  | XM_237293 | Prkag3_predicted | 5.70E-08 | -1.53 |
|  | A_44_P335629 | A_44_P335629 | 8.11E-07 | 1.13 |  |  | CO389876 | CO389876 | 0.001265 | -1.54 |
|  | NM_017201 | Ahcy | 1.67E-06 | 1.13 |  |  | ENSRNOT00000012605 | Unc45b_predicted | 3.38E-08 | -1.54 |
|  | ENSRNOT00000012512 | RGD1563607_predicted | 0.00014 | 1.13 |  |  | NM_013060 | Id2 | 4.42E-07 | -1.54 |
|  | XM_577714 | RGD1565723_predicted | 8.79E-07 | 1.13 |  |  | NM_133619 | Gpha2 | 2.47E-08 | -1.55 |
|  | ENSRNOT00000013962 | Kua_predicted | 6.31E-07 | 1.13 |  |  | ENSRNOT00000059596 | Slu7 | 1.37E-07 | -1.55 |
|  | ENSRNOT00000016714 | Nsun4_predicted | 3.67E-07 | 1.13 |  |  | NM_021696 | Serpinb2 | 0.00057 | -1.55 |
|  | NM_019262 | C1qb | 1.22E-05 | 1.13 |  |  | NM_031831 | Rtn4 | 3.26E-08 | -1.55 |
|  | ENSRNOT00000027173 | Impdh1_predicted | 5.62E-07 | 1.13 |  |  | BQ781487 | BQ781487 | 2.70E-06 | -1.55 |
|  | NM_022847 | Pgr | 1.53E-06 | 1.13 |  |  | ENSRNOT00000017401 | Mtap7_predicted | 1.70E-07 | -1.55 |
|  | BQ202091 | BQ202091 | 3.42E-05 | 1.13 |  |  | ENSRNOT00000024211 | ENSRNOT00000024211 | 0.001214 | -1.55 |
|  | ENSRNOT00000012773 | Ndst4_predicted | 7.17E-06 | 1.13 |  |  | ENSRNOT00000019720 | Ephb6 | 2.14E-07 | -1.55 |
|  | ENSRNOT00000031129 | ENSRNOT00000031129 | 0.000242 | 1.13 |  |  | BM986266 | BM986266 | 5.41E-07 | -1.56 |
|  | ENSRNOT00000043859 | Pde7a | 9.35E-06 | 1.13 |  |  | BI285576 | BI285576 | 6.31E-08 | -1.57 |
|  | NM_001033868 | Surf4 | 2.71E-06 | 1.13 |  |  | NM_130431 | Hspb2 | 2.17E-07 | -1.57 |
|  | AW914848 | AW914848 | 9.07E-07 | 1.13 |  |  | NM_001000327 | Olr575_predicted | 2.05E-07 | -1.57 |
|  | NM_001047878 | F5 | 0.001101 | 1.13 |  |  | ENSRNOT00000002924 | Ccni_predicted | 3.38E-07 | -1.57 |
|  | BF548232 | BF548232 | 0.000456 | 1.12 |  |  | CF111094 | CF111094 | 3.49E-08 | -1.57 |
|  | ENSRNOT00000017078 | Thbs4 | 3.72E-07 | 1.12 |  |  | NM_022215 | Gpd1 | 4.83E-07 | -1.57 |
|  | ENSRNOT00000018535 | Cdh11 | 2.71E-06 | 1.12 |  |  | NM_001017466 | MGC108776 | 3.53E-08 | -1.57 |
|  | NM_031807 | Tpbg | 1.79E-05 | 1.12 |  |  | ENSRNOT00000007593 | Il1f5_predicted | 1.89E-07 | -1.57 |
|  | ENSRNOT00000021433 | Mrpl32_predicted | 3.35E-07 | 1.12 |  |  | NM_017156 | Cyp2b15 | 5.72E-08 | -1.58 |
|  | NM_024160 | Cyba | 4.12E-06 | 1.12 |  |  | NM_001004082 | Hspcb | 2.86E-05 | -1.58 |
|  | ENSRNOT00000051612 | Ttk_predicted | 1.15E-06 | 1.12 |  |  | NM_001002290 | Kprp | 2.23E-08 | -1.58 |
|  | AA955449 | AA955449 | 2.40E-06 | 1.12 |  |  | NM_013130 | Smad1 | 0.001015 | -1.58 |
|  | XM_346040 | Sec61g | 2.43E-06 | 1.12 |  |  | NM_053778 | Ipo13 | 7.78E-07 | -1.58 |
|  | NM_001040180 | Ckap1_predicted | 6.02E-07 | 1.12 |  |  | NM_001047883 | Mrpl49 | 0.000423 | -1.58 |
|  | AF152002 | LOC290595 | 3.54E-07 | 1.12 |  |  | TC615875 | TC615875 | 3.48E-08 | -1.58 |
|  | TC611111 | TC611111 | 0.000177 | 1.12 |  |  | BC086431 | Hdac5 | 5.45E-08 | -1.59 |
|  | NM_017214 | Rgs4 | 1.20E-06 | 1.12 |  |  | NM_053325 | Syt8 | 2.56E-07 | -1.59 |
|  | XM_230899 | Tmepai_predicted | 4.45E-06 | 1.12 |  |  | NM_133582 | Blcap | 7.08E-06 | -1.59 |
|  | AW142634 | AW142634 | 0.000364 | 1.12 |  |  | ENSRNOT00000010007 | Fhl3_predicted | 1.64E-07 | -1.59 |
|  | BM986492 | BM986492 | 5.02E-07 | 1.12 |  |  | NM_053814 | Mrip | 3.84E-06 | -1.59 |
|  | ENSRNOT00000007285 | Mad2l1_predicted | 2.96E-05 | 1.12 |  |  | NM_001005889 | Rdx | 8.51E-06 | -1.59 |
|  | ENSRNOT00000002487 | Rfc4_predicted | 6.69E-06 | 1.12 |  |  | NM_199405 | Lcmt1 | 1.47E-06 | -1.60 |
|  | NM_001009637 | Lars | 4.00E-06 | 1.11 |  |  | XM_345674 | Cfl2_predicted | 6.06E-07 | -1.60 |
|  | NM_053886 | Lman1 | 4.67E-06 | 1.11 |  |  | NM_020104 | Myl1 | 2.52E-07 | -1.60 |
|  | A_44_P627788 | A_44_P627788 | 1.77E-06 | 1.11 |  |  | NM_130423 | Ggtl3 | 2.16E-07 | -1.60 |
|  | NM_173101 | Myo1e | 0.000184 | 1.11 |  |  | NM_012820 | Acsl1 | 4.33E-06 | -1.60 |
|  | NM_173837 | Bbc3 | 2.73E-06 | 1.11 |  |  | DY471696 | DY471696 | 0.000306 | -1.60 |
|  | ENSRNOT00000045710 | ENSRNOT00000045710 | 1.16E-06 | 1.11 |  |  | ENSRNOT00000032324 | Tmem56_predicted | 8.41E-08 | -1.61 |
|  | NM_001024802 | Tparl | 3.12E-06 | 1.11 |  |  | AW143334 | AW143334 | 7.24E-08 | -1.61 |
|  | NM_022381 | Pcna | 2.47E-06 | 1.11 |  |  | NM_001005529 | Ttc1 | 8.15E-05 | -1.61 |
|  | AW917664 | AW917664 | 1.89E-06 | 1.11 |  |  | NM_054006 | Unr | 8.13E-05 | -1.61 |
|  | NM_001004214 | Nqo2 | 2.99E-07 | 1.11 |  |  | BC079339 | Atp6ap2 | 0.001274 | -1.61 |
|  | BG663067 | BG663067 | 6.00E-07 | 1.11 |  |  | NM_012527 | Chrm3 | 2.84E-08 | -1.62 |
|  | ENSRNOT00000059511 | ENSRNOT00000059511 | 5.87E-07 | 1.11 |  |  | ENSRNOT00000055735 | Agl_predicted | 1.32E-07 | -1.62 |
|  | XM_342763 | Fkbp4 | 1.92E-06 | 1.10 |  |  | ENSRNOT00000035566 | ENSRNOT00000035566 | 3.80E-07 | -1.62 |
|  | NM_001033883 | Cxcl12 | 3.11E-07 | 1.10 |  |  | ENSRNOT00000012348 | ENSRNOT00000012348 | 1.15E-07 | -1.62 |
|  | ENSRNOT00000037444 | RGD1310778_predicted | 5.48E-07 | 1.10 |  |  | DV723425 | DV723425 | 6.33E-05 | -1.62 |
|  | XM_233430 | Tal1_predicted | 1.05E-05 | 1.10 |  |  | ENSRNOT00000012373 | Evpl_predicted | 1.67E-08 | -1.62 |
|  | XM_001072896 | Ube2q2_predicted | 4.15E-05 | 1.10 |  |  | ENSRNOT00000026261 | Gup1_predicted | 7.34E-08 | -1.62 |
|  | ENSRNOT00000036738 | Dpy19l1_predicted | 8.77E-05 | 1.10 |  |  | NM_022598 | Cnbp1 | 2.20E-05 | -1.63 |
|  | NM_147177 | Ruvbl1 | 7.82E-07 | 1.10 |  |  | XM_213595 | XM_213595 | 6.36E-07 | -1.63 |
|  | NM_031787 | Hipk3 | 1.04E-05 | 1.10 |  |  | TC585579 | TC585579 | 1.96E-08 | -1.63 |
|  | AF247818 | Tert | 1.50E-06 | 1.10 |  |  | NM_017117 | Capn3 | 2.21E-08 | -1.63 |
|  | ENSRNOT00000017727 | Scube1 | 9.63E-07 | 1.10 |  |  | ENSRNOT00000025758 | Slc25a17_predicted | 6.51E-06 | -1.63 |
|  | ENSRNOT00000044481 | Glrp1_predicted | 1.59E-06 | 1.10 |  |  | NM_053612 | Hspb8 | 8.86E-08 | -1.63 |
|  | NM_001039004 | Cpsf5 | 5.89E-07 | 1.10 |  |  | ENSRNOT00000020812 | Mfap5_predicted | 7.12E-08 | -1.63 |
|  | CO393619 | CO393619 | 0.000529 | 1.10 |  |  | XM_344243 | XM_344243 | 1.89E-07 | -1.63 |
|  | TC613177 | TC613177 | 1.60E-06 | 1.10 |  |  | ENSRNOT00000027893 | Ryr1 | 2.30E-08 | -1.64 |
|  | NM_053592 | Dut | 3.37E-06 | 1.10 |  |  | NM_031983 | Smarcd2 | 4.20E-07 | -1.64 |
|  | NM_053660 | Gng10 | 4.13E-06 | 1.10 |  |  | NM_030987 | Gnb1 | 4.84E-06 | -1.64 |
|  | XM_227418 | XM_227418 | 4.24E-06 | 1.09 |  |  | NM_207617 | Iqsec3 | 3.20E-08 | -1.64 |
|  | ENSRNOT00000012479 | Gas2l1_predicted | 6.07E-07 | 1.09 |  |  | NM_012812 | Cox6a2 | 3.19E-08 | -1.64 |
|  | ENSRNOT00000038162 | ENSRNOT00000038162 | 2.54E-06 | 1.09 |  |  | XM_001071847 | RGD1561490_predicted | 4.53E-06 | -1.64 |
|  | NM_022188 | Robo1 | 2.79E-06 | 1.09 |  |  | NM_022922 | Tpi1 | 2.43E-06 | -1.64 |
|  | ENSRNOT00000018646 | Snrpd1_predicted | 6.83E-07 | 1.09 |  |  | XR_005771 | LOC299727 | 3.30E-07 | -1.64 |
|  | NM_001015014 | Surf6_predicted | 1.01E-06 | 1.09 |  |  | XM_236194 | Mll | 1.90E-05 | -1.64 |
|  | AA875619 | AA875619 | 9.53E-06 | 1.09 |  |  | XR_006381 | LOC302782 | 4.08E-08 | -1.65 |
|  | BC070890 | Sdc2 | 2.55E-05 | 1.09 |  |  | ENSRNOT00000017568 | ENSRNOT00000017568 | 2.39E-06 | -1.65 |
|  | ENSRNOT00000005402 | RGD1561792_predicted | 9.51E-07 | 1.09 |  |  | ENSRNOT00000031577 | ENSRNOT00000031577 | 1.30E-06 | -1.65 |
|  | TC609568 | TC609568 | 3.85E-07 | 1.08 |  |  | NM_019168 | Arg2 | 3.59E-06 | -1.65 |
|  | NM_030834 | Slc16a3 | 8.00E-07 | 1.08 |  |  | NM_031765 | Rxrg | 4.59E-08 | -1.65 |
|  | NM_001037653 | Fubp1 | 1.33E-06 | 1.08 |  |  | CA512164 | CA512164 | 8.67E-06 | -1.65 |
|  | ENSRNOT00000036296 | ENSRNOT00000036296 | 4.59E-06 | 1.08 |  |  | XM_001065536 | Iars2_predicted | 1.49E-07 | -1.65 |
|  | XM_219574 | XM_219574 | 4.85E-07 | 1.08 |  |  | NM_001045843 | MGC112684 | 0.002852 | -1.66 |
|  | TC614636 | TC614636 | 2.69E-06 | 1.08 |  |  | XM_230036 | Ssfa2_predicted | 1.24E-06 | -1.67 |
|  | CF110668 | CF110668 | 0.000222 | 1.08 |  |  | CO394478 | CO394478 | 3.72E-07 | -1.67 |
|  | ENSRNOT00000016410 | Zmpste24_predicted | 0.000107 | 1.08 |  |  | ENSRNOT00000025964 | RGD1560911_predicted | 5.12E-08 | -1.67 |
|  | NM_031977 | Src | 1.06E-06 | 1.08 |  |  | ENSRNOT00000028801 | ENSRNOT00000028801 | 4.53E-06 | -1.67 |
|  | A_44_P330960 | A_44_P330960 | 5.09E-07 | 1.08 |  |  | TC601880 | TC601880 | 2.57E-08 | -1.67 |
|  | BE104250 | BE104250 | 0.000105 | 1.08 |  |  | CB327764 | CB327764 | 9.07E-07 | -1.67 |
|  | AA859130 | AA859130 | 9.39E-07 | 1.08 |  |  | NM_012513 | Bdnf | 7.70E-08 | -1.67 |
|  | NM_001001116 | Olr1720_predicted | 1.05E-05 | 1.08 |  |  | NM_001009692 | Sh3glb2 | 5.54E-08 | -1.68 |
|  | CB316016 | LOC688717 | 4.00E-07 | 1.08 |  |  | AA859994 | AA859994 | 2.97E-08 | -1.68 |
|  | BU946539 | BU946539 | 2.93E-06 | 1.08 |  |  | XR_009063 | RGD1560581_predicted | 1.73E-08 | -1.68 |
|  | BC099783 | BC099783 | 2.56E-06 | 1.08 |  |  | ENSRNOT00000008904 | RGD1561831_predicted | 7.99E-08 | -1.68 |
|  | NM_022229 | Hspd1 | 7.61E-06 | 1.08 |  |  | NM_001010970 | Amy1 | 6.55E-05 | -1.68 |
|  | NM_001006955 | Cldnd1 | 3.59E-06 | 1.07 |  |  | DV723417 | DV723417 | 1.72E-06 | -1.68 |
|  | XM_215278 | Wbp5_predicted | 1.52E-06 | 1.07 |  |  | NM_001007608 | Skp1a | 0.003605 | -1.69 |
|  | ENSRNOT00000037028 | Kif23_predicted | 4.39E-07 | 1.07 |  |  | A_44_P821386 | A_44_P821386 | 8.10E-07 | -1.69 |
|  | ENSRNOT00000029014 | ENSRNOT00000029014 | 0.000251 | 1.07 |  |  | ENSRNOT00000013286 | RGD1305081_predicted | 1.20E-07 | -1.69 |
|  | AI411122 | AI411122 | 1.61E-06 | 1.07 |  |  | NM_175843 | Sqstm1 | 1.60E-07 | -1.69 |
|  | XM_342828 | Melk_predicted | 1.97E-05 | 1.07 |  |  | NM_013113 | Atp1b1 | 7.18E-08 | -1.70 |
|  | NM_019139 | Gdnf | 7.37E-05 | 1.07 |  |  | NM_031721 | Htra1 | 3.26E-06 | -1.70 |
|  | ENSRNOT00000018025 | Mib1_predicted | 8.80E-07 | 1.07 |  |  | NM_001004078 | Cct5 | 5.65E-05 | -1.70 |
|  | XM_001066326 | Rp1l1_predicted | 1.24E-06 | 1.07 |  |  | A_44_P536873 | A_44_P536873 | 2.67E-07 | -1.70 |
|  | NM_030858 | Smad7 | 5.32E-07 | 1.07 |  |  | NM_021746 | Mapk12 | 6.92E-08 | -1.70 |
|  | TC586420 | TC586420 | 1.84E-06 | 1.07 |  |  | ENSRNOT00000009903 | Smtnl1_predicted | 1.66E-08 | -1.70 |
|  | NM_017210 | Dio3 | 5.91E-07 | 1.07 |  |  | NM_012943 | Dlx5 | 4.38E-08 | -1.70 |
|  | NM_021765 | Copb2 | 1.16E-06 | 1.07 |  |  | NM_031007 | Adcy2 | 1.76E-08 | -1.70 |
|  | BF417038 | BF417038 | 0.000118 | 1.06 |  |  | XR_008863 | LOC499693 | 3.78E-06 | -1.71 |
|  | ENSRNOT00000059486 | ENSRNOT00000059486 | 7.71E-05 | 1.06 |  |  | NM_001012218 | Rnf29 | 3.07E-08 | -1.71 |
|  | XM_001078547 | LOC691499 | 3.22E-05 | 1.06 |  |  | ENSRNOT00000003495 | Serpinb11_predicted | 0.002573 | -1.71 |
|  | A_44_P982920 | A_44_P982920 | 0.001011 | 1.06 |  |  | ENSRNOT00000057129 | Sesn1_predicted | 8.98E-08 | -1.71 |
|  | BF389721 | BF389721 | 0.001673 | 1.06 |  |  | NM_031043 | Gyg1 | 0.005253 | -1.71 |
|  | BI283060 | BI283060 | 1.39E-06 | 1.06 |  |  | NM_001013942 | Rsnl2 | 1.29E-08 | -1.71 |
|  | TC636700 | TC636700 | 1.46E-06 | 1.05 |  |  | ENSRNOT00000020673 | RGD1306073_predicted | 2.69E-08 | -1.71 |
|  | NM_001024345 | MGC109519 | 4.51E-06 | 1.05 |  |  | ENSRNOT00000001196 | Rabgef1_predicted | 1.48E-05 | -1.71 |
|  | NM_181477 | Prss21 | 1.34E-05 | 1.05 |  |  | BF544796 | BF544796 | 1.77E-07 | -1.72 |
|  | ENSRNOT00000027867 | Krtcap2_predicted | 5.69E-06 | 1.05 |  |  | ENSRNOT00000008159 | RGD1562562_predicted | 1.16E-07 | -1.72 |
|  | AI171962 | AI171962 | 1.25E-05 | 1.05 |  |  | NM_133618 | Hadhb | 6.21E-05 | -1.72 |
|  | XM_213425 | Ccl12_predicted | 2.37E-06 | 1.05 |  |  | NM_053515 | Slc25a4 | 2.27E-07 | -1.72 |
|  | XM_342569 | XM_342569 | 2.75E-06 | 1.05 |  |  | XR_008228 | RGD1566399_predicted | 4.74E-05 | -1.72 |
|  | CO574382 | CO574382 | 3.15E-06 | 1.05 |  |  | XM_229646 | XM_229646 | 3.50E-06 | -1.72 |
|  | AA997322 | AA997322 | 4.08E-06 | 1.05 |  |  | NM_021767 | Nrxn1 | 1.13E-08 | -1.72 |
|  | ENSRNOT00000031583 | RGD1305975_predicted | 5.16E-07 | 1.05 |  |  | XM_001070764 | LOC689427 | 2.57E-08 | -1.73 |
|  | AW915403 | AW915403 | 1.27E-06 | 1.05 |  |  | NM_198765 | Bicd2 | 0.000343 | -1.73 |
|  | ENSRNOT00000044768 | LOC289786 | 2.01E-06 | 1.05 |  |  | NM_001034129 | Tloc1 | 0.001306 | -1.73 |
|  | ENSRNOT00000024719 | Urod | 6.15E-07 | 1.05 |  |  | XR_005944 | LOC681019 | 4.15E-08 | -1.74 |
|  | ENSRNOT00000057073 | Lrfn1_predicted | 1.43E-06 | 1.05 |  |  | ENSRNOT00000054917 | Rab40b_predicted | 2.41E-08 | -1.74 |
|  | NM_001014097 | LOC315970 | 1.31E-06 | 1.05 |  |  | NM_053819 | Timp1 | 0.000551 | -1.74 |
|  | XM_341882 | Sytl2_predicted | 7.64E-06 | 1.05 |  |  | ENSRNOT00000026920 | ENSRNOT00000026920 | 1.47E-05 | -1.74 |
|  | NM_012814 | Cox6a1 | 1.05E-06 | 1.05 |  |  | NM_080903 | Trim63 | 9.77E-09 | -1.74 |
|  | NM_013053 | Ywhaq | 5.53E-06 | 1.05 |  |  | XM_345912 | RGD1564209_predicted | 3.27E-06 | -1.75 |
|  | NM_024483 | Adra1d | 6.18E-06 | 1.05 |  |  | NM_031031 | Gatm | 4.16E-08 | -1.75 |
|  | ENSRNOT00000061379 | LOC688946 | 7.67E-07 | 1.04 |  |  | ENSRNOT00000037432 | RGD1559590_predicted | 1.21E-08 | -1.75 |
|  | ENSRNOT00000032433 | Glis1_predicted | 6.54E-06 | 1.04 |  |  | NM_182671 | LOC316632 | 3.40E-06 | -1.76 |
|  | TC596940 | TC596940 | 5.24E-07 | 1.04 |  |  | NM_139333 | Prpf19 | 1.77E-05 | -1.76 |
|  | NM_001013931 | RGD1311249 | 1.15E-06 | 1.04 |  |  | NM_031750 | Hspb3 | 3.34E-08 | -1.76 |
|  | TC604161 | TC604161 | 1.52E-06 | 1.04 |  |  | ENSRNOT00000008229 | ENSRNOT00000008229 | 0.000311 | -1.76 |
|  | NM_001012142 | Scrn2 | 4.85E-05 | 1.04 |  |  | XM_233065 | RGD1561090_predicted | 2.27E-07 | -1.77 |
|  | ENSRNOT00000025071 | Sox4_predicted | 0.013953 | 1.04 |  |  | ENSRNOT00000024587 | Dusp22_predicted | 1.18E-05 | -1.77 |
|  | BF396146 | S100a4 | 1.16E-05 | 1.04 |  |  | BQ194442 | BQ194442 | 2.03E-07 | -1.77 |
|  | ENSRNOT00000018527 | RGD1311752_predicted | 1.86E-06 | 1.04 |  |  | AW921086 | AW921086 | 1.55E-08 | -1.78 |
|  | XM_343674 | LOC363336 | 6.36E-07 | 1.04 |  |  | NM_001012160 | Gkap1 | 3.55E-06 | -1.78 |
|  | ENSRNOT00000001464 | ENSRNOT00000001464 | 2.73E-06 | 1.04 |  |  | XR_005506 | LOC362250 | 1.00E-07 | -1.79 |
|  | ENSRNOT00000019761 | Pigt_predicted | 5.83E-07 | 1.04 |  |  | NM_021663 | Nucb2 | 0.000173 | -1.79 |
|  | A_44_P494413 | A_44_P494413 | 7.64E-05 | 1.04 |  |  | NM_134364 | Atp5b | 2.78E-06 | -1.79 |
|  | NM_031032 | Gmfb | 0.000229 | 1.04 |  |  | ENSRNOT00000016663 | ENSRNOT00000016663 | 4.50E-06 | -1.79 |
|  | XM_001055332 | Pctk2 | 1.05E-06 | 1.03 |  |  | XM_230036 | Ssfa2_predicted | 9.86E-05 | -1.79 |
|  | ENSRNOT00000036343 | RGD1305254_predicted | 7.64E-07 | 1.03 |  |  | A_44_P459484 | A_44_P459484 | 7.82E-09 | -1.79 |
|  | AA956502 | AA956502 | 4.87E-05 | 1.03 |  |  | ENSRNOT00000027530 | Taf13_predicted | 0.000551 | -1.79 |
|  | NM_031535 | Bcl2l1 | 1.49E-05 | 1.03 |  |  | AA926010 | AA926010 | 9.79E-08 | -1.80 |
|  | NM_031129 | Tceb2 | 3.50E-05 | 1.03 |  |  | XR_006415 | LOC688528 | 1.16E-08 | -1.80 |
|  | NM_024125 | Cebpb | 4.77E-07 | 1.03 |  |  | DV728647 | DV728647 | 4.65E-06 | -1.80 |
|  | BE118459 | BE118459 | 0.000151 | 1.03 |  |  | NM_053871 | Srp54 | 0.000289 | -1.80 |
|  | ENSRNOT00000006205 | Nxt1_predicted | 6.88E-07 | 1.03 |  |  | NM_020540 | Gstm4 | 8.14E-08 | -1.80 |
|  | AI408948 | AI408948 | 3.26E-06 | 1.03 |  |  | XM_340999 | Tfrc | 0.002827 | -1.81 |
|  | BQ208553 | BQ208553 | 8.25E-06 | 1.03 |  |  | NM_001004022 | Ka15 | 1.50E-08 | -1.81 |
|  | NM_199395 | Geft | 1.75E-06 | 1.03 |  |  | NM_001013889 | LOC291863 | 4.23E-06 | -1.81 |
|  | NM_133598 | Gcsh | 4.99E-06 | 1.03 |  |  | ENSRNOT00000026219 | Lipl3_predicted | 0.000134 | -1.81 |
|  | ENSRNOT00000011158 | Nipsnap1 | 1.76E-06 | 1.03 |  |  | NM_012502 | Ar | 1.95E-07 | -1.81 |
|  | NM_138838 | Pou3f1 | 1.23E-06 | 1.02 |  |  | NM_001010963 | LOC362154 | 0.003891 | -1.82 |
|  | NM_001002850 | Prm1 | 0.000181 | 1.02 |  |  | ENSRNOT00000016357 | ENSRNOT00000016357 | 3.45E-07 | -1.82 |
|  | TC603506 | TC603506 | 1.54E-06 | 1.02 |  |  | NM_130406 | Faf1 | 3.53E-06 | -1.83 |
|  | NM_057102 | Slc25a5 | 4.36E-05 | 1.02 |  |  | NM_019334 | Pitx2 | 9.25E-09 | -1.83 |
|  | XM_218516 | Zfp537_predicted | 5.98E-07 | 1.02 |  |  | NM_012891 | Acadvl | 7.96E-07 | -1.83 |
|  | NM_022236 | Pde10a | 9.14E-07 | 1.02 |  |  | ENSRNOT00000002980 | RGD1310958_predicted | 2.34E-08 | -1.84 |
|  | TC591246 | TC591246 | 1.17E-05 | 1.02 |  |  | ENSRNOT00000004638 | RGD1306729_predicted | 1.49E-08 | -1.84 |
|  | NM_001024887 | Sigirr | 1.86E-06 | 1.02 |  |  | NM_024349 | Ak1 | 1.64E-06 | -1.84 |
|  | XM_001077741 | LOC687259 | 1.33E-06 | 1.02 |  |  | NM_001079893 | Dusp14_predicted | 4.62E-06 | -1.84 |
|  | NM_012615 | Odc1 | 2.02E-06 | 1.02 |  |  | NM_130734 | Gnb2l1 | 4.61E-06 | -1.84 |

P value correction was done using Benjamini and Hochberg method.

**Supplementary table 2.** Differentially expressed genes in DMBA+chlorophyllin treated hamsters (P=0.05, fold change cut off- 2).

P value correction was done using Benjamini and Hochberg method.

P value correction was done using Benjamini and Hochberg method.

P value correction was done using Benjamini and Hochberg method.
