# Supplementary material for: Gene Expression Signature of DMBA-Induced Hamster Buccal Pouch Carcinomas: Modulation by Chlorophyllin and Ellagic Acid
Source: PLoS One. 2012 Apr 2;7(4):e34628. doi: 10.1371/journal.pone.0034628 (PMC3317635; doi:10.1371/journal.pone.0034628)
Supplement: Table S2 — List of differentially expressed genes in DMBA+chlorophyllin treated hamsters (P = 0.05, fold change cut off- 2). (DOC) [file pone.0034628.s002.doc]

| **S.No** | **Systematic name** | **Gene name** | **P value** | **Fold change** |  | **S.No** | **Systematic name** | **Gene name** | **P value** | **Fold change** |
| --- | --- | --- | --- | --- | --- | --- | --- | --- | --- | --- |
|  | NM_001024866 | RGD1311732 | 2.42E-05 | 2.71 |  |  | XM_001077843 | Chl1 | 5.33E-07 | 1.10 |
|  | NM_001000164 | Olr144_predicted | 0.000102 | 2.45 |  |  | NM_145682 | Filip1 | 8.85E-07 | 1.10 |
|  | TC592522 | TC592522 | 1.04E-05 | 2.34 |  |  | CF109666 | CF109666 | 2.39E-05 | 1.09 |
|  | CB557922 | CB557922 | 0.00044 | 2.21 |  |  | XM_220534 | Usp22_predicted | 8.16E-06 | 1.09 |
|  | ENSRNOT00000000155 | Plxdc2_predicted | 1.78E-05 | 2.21 |  |  | ENSRNOT00000021228 | Prkrir_predicted | 7.03E-05 | 1.09 |
|  | TC575338 | TC575338 | 3.25E-09 | 2.20 |  |  | XM_345817 | XM_345817 | 0.004175 | 1.08 |
|  | ENSRNOT00000006952 | RGD1562576_predicted | 0.000216 | 2.13 |  |  | NM_001047087 | Eif3s10 | 1.41E-06 | 1.08 |
|  | ENSRNOT00000058611 | RGD1305797_predicted | 9.03E-05 | 2.10 |  |  | AI535429 | AI535429 | 2.38E-06 | 1.08 |
|  | ENSRNOT00000024968 | Cidea_predicted | 0.001037 | 2.10 |  |  | TC632166 | TC632166 | 0.003241 | 1.07 |
|  | NM_053294 | Adora2a | 0.001418 | 2.10 |  |  | TC611111 | TC611111 | 0.000198 | 1.07 |
|  | NM_012699 | Dnajb9 | 8.25E-06 | 2.04 |  |  | NM_012923 | Ccng1 | 0.000157 | 1.06 |
|  | ENSRNOT00000038863 | RGD1304731_predicted | 1.69E-07 | 2.04 |  |  | TC631080 | TC631080 | 0.000248 | 1.04 |
|  | NM_022213 | Pik3r3 | 2.50E-05 | 1.99 |  |  | ENSRNOT00000012512 | RGD1563607_predicted | 0.000198 | 1.04 |
|  | ENSRNOT00000001882 | Sdsl_predicted | 8.00E-05 | 1.82 |  |  | NM_030866 | Nfix | 1.26E-05 | 1.04 |
|  | TC605360 | TC605360 | 0.00022 | 1.78 |  |  | ENSRNOT00000037389 | Myst3 | 5.17E-05 | 1.03 |
|  | BF391602 | RGD1559578_predicted | 2.77E-06 | 1.75 |  |  | AW251931 | AW251931 | 9.59E-05 | 1.02 |
|  | BE099798 | BE099798 | 3.89E-07 | 1.72 |  |  | XM_215666 | Trim45_predicted | 6.31E-06 | 1.01 |
|  | XM_240367 | RGD1563825_predicted | 0.000172 | 1.70 |  |  | NM_013088 | Ptpn11 | 1.15E-06 | 1.00 |
|  | BE119385 | BE119385 | 0.001105 | 1.68 |  |  | M61725 | Ubtf | 5.52E-05 | 1.00 |
|  | CB548031 | CB548031 | 0.000413 | 1.59 |  |  | BE109644 | BE109644 | 0.001124 | 0.99 |
|  | BE111887 | BE111887 | 0.001922 | 1.56 |  |  | NM_145721 | Cdk5rap1 | 0.003297 | 0.99 |
|  | XM_576094 | RGD1560932_predicted | 7.56E-05 | 1.56 |  |  | NM_032079 | Dnaja2 | 0.001232 | 0.99 |
|  | TC605762 | TC605762 | 0.000121 | 1.49 |  |  | AA963226 | AA963226 | 2.57E-06 | -0.98 |
|  | NM_013066 | Mtap2 | 2.58E-07 | 1.49 |  |  | BM390457 | BM390457 | 4.62E-05 | -0.99 |
|  | ENSRNOT00000036748 | Wdr41_predicted | 1.05E-05 | 1.48 |  |  | BG153272 | BG153272 | 3.87E-06 | -1.00 |
|  | BI282039 | BI282039 | 0.0003 | 1.40 |  |  | CB547064 | CB547064 | 0.003397 | -1.02 |
|  | TC624759 | TC624759 | 0.001156 | 1.37 |  |  | AA997406 | AA997406 | 9.06E-06 | -1.0 |
|  | NM_031736 | Slc27a2 | 0.000127 | 1.36 |  |  | ENSRNOT00000043415 | ENSRNOT00000043415 | 1.08E-05 | -1.05 |
|  | BQ199904 | BQ199904 | 4.76E-05 | 1.35 |  |  | BM986596 | BM986596 | 2.12E-05 | -1.05 |
|  | XM_346956 | RGD1564528_predicted | 8.26E-05 | 1.35 |  |  | AA997829 | AA997829 | 0.000165 | -1.05 |

**Table S2.** Differentially expressed genes in DMBA+chlorophyllin treated hamsters (P=0.05, fold change cut off- 2).

P value correction was done using Benjamini and Hochberg method.

| **S.No** | **Systematic name** | **Gene name** | **P value** | **Fold change** |  | **S.No** | **Systematic name** | **Gene name** | **P value** | **Fold change** |
| --- | --- | --- | --- | --- | --- | --- | --- | --- | --- | --- |
|  | BE119393 | BE119393 | 0.000537 | 1.34 |  |  | AY539882 | LOC500845 | 1.93E-06 | -1.06 |
|  | NM_001044244 | Tomm34_predicted | 2.07E-05 | 1.31 |  |  | AABR03058881 | AABR03058881 | 7.31E-06 | -1.06 |
|  | NM_031982 | Trpv1 | 0.000157 | 1.30 |  |  | BF284253 | BF284253 | 4.78E-06 | -1.06 |
|  | AA800053 | AA800053 | 7.67E-06 | 1.30 |  |  | AA945841 | AA945841 | 8.12E-07 | -1.06 |
|  | NM_001013170 | Wars | 7.39E-07 | 1.29 |  |  | BF289687 | BF289687 | 4.98E-07 | -1.06 |
|  | ENSRNOT00000020524 | Nt5c1a_predicted | 0.002638 | 1.28 |  |  | A_44_P631361 | A_44_P631361 | 1.01E-06 | -1.08 |
|  | NM_001047878 | F5 | 0.000537 | 1.27 |  |  | AW525193 | AW525193 | 1.09E-05 | -1.08 |
|  | AA925529 | AA925529 | 0.001251 | 1.27 |  |  | AA848453 | AA848453 | 9.96E-07 | -1.10 |
|  | BF389721 | BF389721 | 0.000618 | 1.26 |  |  | BQ192759 | BQ192759 | 6.66E-06 | -1.10 |
|  | A_44_P347431 | A_44_P347431 | 4.29E-05 | 1.26 |  |  | AABR03004099 | AABR03004099 | 3.34E-06 | -1.10 |
|  | CR753932 | CR753932 | 1.51E-05 | 1.25 |  |  | NM_001024332 | LOC500282 | 0.001398 | -1.12 |
|  | A_44_P394031 | A_44_P394031 | 0.001541 | 1.23 |  |  | BQ200408 | BQ200408 | 1.94E-06 | -1.12 |
|  | ENSRNOT00000056944 | RGD1311920_predicted | 1.65E-06 | 1.20 |  |  | AA799582 | AA799582 | 3.28E-05 | -1.14 |
|  | XM_001062341 | RGD1560755_predicted | 0.000119 | 1.19 |  |  | BE111604 | BE111604 | 5.43E-07 | -1.15 |
|  | CA506147 | CA506147 | 5.59E-06 | 1.18 |  |  | A_44_P766528 | A_44_P766528 | 1.65E-07 | -1.19 |
|  | NM_012978 | Lhcgr | 2.66E-05 | 1.17 |  |  | BQ198742 | BQ198742 | 2.67E-06 | -1.23 |
|  | ENSRNOT00000043702 | ENSRNOT00000043702 | 0.000157 | 1.16 |  |  | AW920871 | AW920871 | 3.58E-07 | -1.24 |
|  | CO393619 | CO393619 | 0.000383 | 1.16 |  |  | AABR03055433 | AABR03055433 | 2.01E-07 | -1.27 |
|  | XM_235023 | RGD1560606_predicted | 1.95E-05 | 1.16 |  |  | NM_021578 | Tgfb1 | 3.81E-06 | -1.28 |
|  | ENSRNOT00000059463 | Neb_predicted | 2.93E-05 | 1.12 |  |  | BM383877 | BM383877 | 2.28E-05 | -1.33 |
|  | U18650 | Hdh | 0.000236 | 1.12 |  |  | NM_033234 | Hbb | 7.97E-06 | -1.34 |
|  | BI297059 | BI297059 | 0.000199 | 1.11 |  |  | AA859178 | AA859178 | 1.55E-06 | -1.38 |

**Supplementary table 2.** Differentially expressed genes in DMBA+chlorophyllin treated hamsters (P=0.05, fold change cut off- 2).

P value correction was done using Benjamini and Hochberg method.
