# Supplementary material for: Gene Expression Signature of DMBA-Induced Hamster Buccal Pouch Carcinomas: Modulation by Chlorophyllin and Ellagic Acid
Source: PLoS One. 2012 Apr 2;7(4):e34628. doi: 10.1371/journal.pone.0034628 (PMC3317635; doi:10.1371/journal.pone.0034628)
Supplement: Table S3 — List of differentially expressed genes in DMBA+ellagic acid treated hamsters (P = 0.05, fold change cut off- 2). (DOC) [file pone.0034628.s003.doc]

P value correction was done using Benjamini and Hochberg method.

**Table S3.** Differentially expressed genes in DMBA+ellagic acid treated hamsters (P=0.05, fold change cut off- 2).

**Supplementary table 2.** Differentially expressed genes in DMBA+chlorophyllin treated hamsters (P=0.05, fold change cut off- 2).

P value correction was done using Benjamini and Hochberg method.

| **S.No** | **Systematic name** | **Gene name** | **P value** | **Fold change** |  | **S.No** | **Systematic name** | **Gene name** | **P value** | **Fold change** |
| --- | --- | --- | --- | --- | --- | --- | --- | --- | --- | --- |
|  | NM_001024866 | RGD1311732 | 0.000118 | 2.12 |  |  | CA506147 | CA506147 | 8.90E-06 | 1.10 |
|  | TC575338 | TC575338 | 9.41E-09 | 1.89 |  |  | ENSRNOT00000042208 | Ccdc58_predicted | 1.07E-06 | 1.10 |
|  | TC592522 | TC592522 | 9.70E-05 | 1.66 |  |  | ENSRNOT00000003973 | Cdx4_predicted | 1.06E-05 | 1.1 |
|  | NM_022213 | Pik3r3 | 0.000153 | 1.50 |  |  | NM_012562 | Fuca | 0.000677 | 1.08 |
|  | AA925529 | AA925529 | 0.000625 | 1.43 |  |  | ENSRNOT00000002156 | Evx2_predicted | 0.00091 | 1.07 |
|  | CR753932 | CR753932 | 9.55E-06 | 1.34 |  |  | NM_022293 | Kcnk13 | 7.12E-05 | 1.07 |
|  | NM_001013170 | Wars | 6.26E-07 | 1.32 |  |  | NM_031736 | Slc27a2 | 0.000639 | 1.04 |
|  | CB548031 | CB548031 | 0.001442 | 1.28 |  |  | XM_219680 | RGD1306343_predicted | 7.73E-06 | 1.02 |
|  | BI282039 | BI282039 | 0.000504 | 1.28 |  |  | NM_013066 | Mtap2 | 4.01E-06 | 1.00 |
|  | XM_346956 | RGD1564528_predicted | 0.000118 | 1.27 |  |  | NM_021578 | Tgfb1 | 3.36E-05 | -0.96 |
|  | XM_215666 | Trim45_predicted | 1.71E-06 | 1.26 |  |  | ENSRNOT00000005222 | Ap3s1_predicted | 6.90E-07 | -1.00 |
|  | AW251931 | AW251931 | 4.64E-05 | 1.18 |  |  | AA997406 | AA997406 | 1.38E-05 | -1.00 |
|  | NM_012699 | Dnajb9 | 0.000342 | 1.14 |  |  | DV721486 | DV721486 | 0.000915 | -1.01 |
|  | BF391602 | RGD1559578_predicted | 4.86E-05 | 1.14 |  |  | BF289687 | BF289687 | 2.63E-06 | -1.08 |
|  | XM_215376 | Mcm3ap_predicted | 7.34E-07 | 1.14 |  |  | BM383877 | BM383877 | 0.000109 | -1.08 |
|  | ENSRNOT00000000155 | Plxdc2_predicted | 0.001124 | 1.14 |  |  | AW920871 | AW920871 | 1.09E-06 | -1.10 |
|  | ENSRNOT00000001882 | Sdsl_predicted | 0.001462 | 1.13 |  |  | AA799582 | AA799582 | 4.85E-05 | -1.11 |
|  | XM_235023 | RGD1560606_predicted | 2.59E-05 | 1.11 |  |  | AA899326 | AA899326 | 4.52E-05 | -1.15 |
|  | NM_012978 | Lhcgr | 3.80E-05 | 1.11 |  |  |  |  |  |  |
